# Autism spectrum disorders, endocrine disrupting compounds, and heavy metals in amniotic fluid: a case-control study

**DOI:** 10.1186/s13229-018-0253-1

**Published:** 2019-01-09

**Authors:** Manhai Long, Mandana Ghisari, Lisbeth Kjeldsen, Maria Wielsøe, Bent Nørgaard-Pedersen, Erik Lykke Mortensen, Morsi W. Abdallah, Eva C. Bonefeld-Jørgensen

**Affiliations:** 1 Centre for Arctic Health & Molecular Epidemiology, Department of Public Health, Aarhus University, Aarhus, Denmark; 2 Section of Neonatal Genetic, Danish Centre for Neonatal Screening, Statens Serum Institute, Copenhagen, Denmark; 3 Section of Environmental Health, Department of Public Health, University of Copenhagen, Copenhagen, Denmark; 4 Department of Child and Adolescent Psychiatry Hofheim, Vitos Psychiatric Hospital Riedstadt, Riedstadt, Germany; 5 Greenland Center for Health Research, Greenland University, Nuuk, Greenland

**Keywords:** Autism, Endocrine disrupting compounds, Receptor activity, Amniotic fluid

## Abstract

**Background:**

Evidence has indicated that some non-inherited factors such as exposure to environmental pollutants are associated with neurodevelopment disorders like autism spectrum disorder (ASD). Studies report that endocrine disrupting compounds (EDCs), including polychlorinated biphenyls, organochlorine pesticides, perfluoroalkyl substances (PFAS), and some metals, have adverse effects on the fetal neurodevelopment. The aim of this study was to measure the amniotic fluid (AF) levels of EDCs and metals as well as the receptor transactivities induced by AF and investigate the possible link between prenatal exposure to EDCs and heavy metals and ASD risk.

**Methods:**

In this case-control study, we included AF samples of 75 ASD cases and 135 frequency-matched controls and measured the levels of the endogenous sex hormones, PFAS, and elements including heavy metals. The combined effect of endogenous hormones and EDCs on the receptor of estrogen (ER), androgen (AR), aryl hydrocarbon (AhR), and thyroid hormone-like activity were also determined and expressed as receptor ligand equivalents. We assessed the associations of AF levels of chemicals, sex hormones, and receptor activities with ASD risk using unconditional logistical regression analyses. To control for multiple comparisons, the false discovery rate (FDR) was used and *q* values less than 0.25 were designated as statistical significance.

**Results:**

PFAS and metals were detectable in AF samples. The ASD cases had significantly lower AF levels of PFAS than controls, and the adjusted odds ratio (OR) was 0.410 (95% CI 0.174, 0.967; *p* = 0.042; FDR *q*_value_ = 0.437) for perfluorooctane sulfonate (PFOS). The principal component, including PFAS congeners, copper, iron, and estrogenic activity, was significantly inversely associated with ASD risk (adjusted OR = 0.100; 95% CI 0.016, 0.630; *p* = 0.014; FDR *q*_value_ = 0.098).

Testosterone level in AF weakly associated with ASD risk (adjusted OR = 1.002; 95% CI 1.000, 1.004; *p* = 0.05). However, after multiple comparison correction, the association was not significant (FDR *q*_value_ = 0.437). No significant associations between AF-induced receptor transactivities and ASD risk were observed. The adjusted OR was 2.176 (95%CI 0.115, 41.153) for the ratio of the combined androgenic activity to combined estrogenic activity.

**Conclusions:**

The presence of PFAS and heavy metals in AF indicates that they can cross the placenta. The inverse association between levels of PFAS congeners in AF and ASD risk might relate to the weak estrogenic activities and anti-androgenic activities of PFAS.

The observed tendency of positive association between the ratio of combined androgenic effect to the combined estrogenic effect and ASD risk needs further studies to explore whether EDCs together with endogenous hormones play a role in the development of ASD.

**Electronic supplementary material:**

The online version of this article (10.1186/s13229-018-0253-1) contains supplementary material, which is available to authorized users.

## Background

Autism spectrum disorder (ASD) is a group of heterogeneous neurodevelopmental disorders characterized by qualitative impairments in social interaction, communication, and repetitive or stereotyped behavior [1]. Epidemiological studies indicate an increasing worldwide prevalence of ASD during the last decades [2, 3]. In Denmark, prevalence of ASD has increased between 1972 and 2007 and a recent estimate was approximately 0.74% [4].

The etiology of ASD is not fully clarified. Despite the evidence of a genetic component [5], genetic factors cannot fully explain ASD, and single gene and chromosomal defects only account for a minority of ASD cases (10–20%) [6, 7]. The accumulating evidence shows that some non-inherited factors such as exposure to environmental pollutants and neurotoxins are associated with ASD risk [8–11]. Moreover, studies suggest that the endocrine system plays a role in the etiology of ASD [12]. Autistic traits were increased following prenatal exposure to abnormally high levels of testosterone caused by congenital adrenal hyperplasia [13]. ASD was linked with elevated fetal testosterone levels in amniotic fluid during routine amniocentesis [14]. A study in 192 twins reported that environmental factors accounted for 55% of the risk of developing ASD compared with genetic factors accounting for 37% [15]. One review reported that 89% of the included publications implicated an association between environmental toxicants and ASD [16].

Children are vulnerable to the neurotoxic effects of chemicals, especially during the prenatal period, when there is an immature blood-brain barrier and neuronal growth, migration, and myelination processes that occur on a specific and rapid schedule [17, 18]. Moreover, some toxic substances can pass through the placental barrier and easily access the developing brain and interfere with these important processes, possibly leading to adverse outcomes [17]. Some environmental pollutants can interfere with the endocrine system acting as endocrine disrupting compounds (EDCs), including persistent organic pollutants (POPs) such as polychlorinated biphenyls (PCBs), organochlorine pesticides (OCPs), polybrominated diphenyl ethers (PBDEs), dioxins [19–22], perfluoroalkyl substances (PFAS) [23, 24], and non-persistent compounds like bisphenol A [25–27].

EDCs may alter endogenous axes and interfere with steroid-dependent neurodevelopment and modify the ASD risk [28, 29]. A variety of EDCs in blood and urine samples of pregnant women were reported to be related to children’s autistic behavior [12]. Animal studies reported that PFAS had adverse effects on neurodevelopment [30, 31]. A Korean study showed that mothers of children with ASD had higher exposure to PBDEs, PCBs, bisphenol A, and dioxins through canned food, plastics, waste incinerators, old electronics, microwavable food, and textiles compared to mothers with typically developing children [32]. Studies have linked autistic behavior with prenatal exposure to the organophosphate insecticide chlorpyrifos [33] and phthalates [34]. In addition, a study reported that gestational exposure to the OCPs, dicofol and endosulfan, was associated with an increased risk of ASD [9]. Parental occupational exposure to chemicals such as solvent xylene was reported to be associated with ASD [35]. Some elements and heavy metals, such as iron (Fe), copper (Cu), zinc (Zn), selenium (Se), iodine (I), arsenic (As), mercury (Hg), cadmium (Cd), lead (Pb), and manganese (Mn), might impact on the development of the nervous system [29, 36–41]. Perinatal exposure to air pollutants, including Hg, Pb, and Mn, was linked to an increased risk of ASD with stronger associations for boys [42].

Humans are exposed to dozens of potential EDCs across the lifespan. The fetus and infant have relatively greater exposure to some EDCs and are more vulnerable than adults. Multiple EDCs may have a combined effect, and the health-related consequences of EDC mixtures are thus of concern. An approach to assess the combined EDC effects of complex EDC mixtures is to measure the overall impact of all EDCs present in a sample on the hormone receptor-mediated transactivation such as measurement of estrogen receptor (ER) transactivity, androgen receptor (AR) transactvity, thyroid hormone (TH) function, and aryl hydrocarbon receptor (AhR) transactivity in the biological samples using ex vivo mammalian cell culture systems [43, 44].

The matrices for prenatal exposure biomarkers are often maternal blood and umbilical cord blood, but correlations with measures in fetal compartments can be low. In practice, umbilical cord blood is obtainable only at birth, but the expected vulnerable time window for many health outcomes is often earlier in the pregnancy (e.g., during organogenesis). Amniotic fluid (AF) offers another possible fetal matrix, but samples are most often only available from amniocentesis indicated by advanced maternal age or by prenatal diagnostics of chromosomal abnormalities and severe malformations. It is a major advantage, however, that amniocentesis is usually performed within the second trimester (gestational weeks 13 to 28), which may be closer to the expected vulnerable period for many adverse outcomes. It is suggested that contaminant concentrations in AF during the first half of pregnancy (before 20 weeks of gestation) can be a suitable proxy measure of intra-fetal contaminant exposure, because AF is derived mainly from fetal blood and fluids during this period [45]. Studies have shown that the risk of autism tends to be higher upon exposures within the first half of pregnancy [46]. Although contaminants in maternal blood or umbilical cord blood are used as a proxy for fetal exposure, little is known about the actual exposure level of the fetus.

The purpose of this study was using AF to (1) measure EDCs and elements including heavy metals, (2) determine the receptor transactivities induced by EDCs, and (3) perform a case-control study to investigate the hypothesis that in utero exposure to EDCs and heavy metals is associated with an increased risk for ASD. In addition, principle component analysis was used to identify the potential underlying components of substances in AF to explore the effect of substance mixture on ASD risk.

## Methods

### Study population and sample selection

**Fig. 1:**
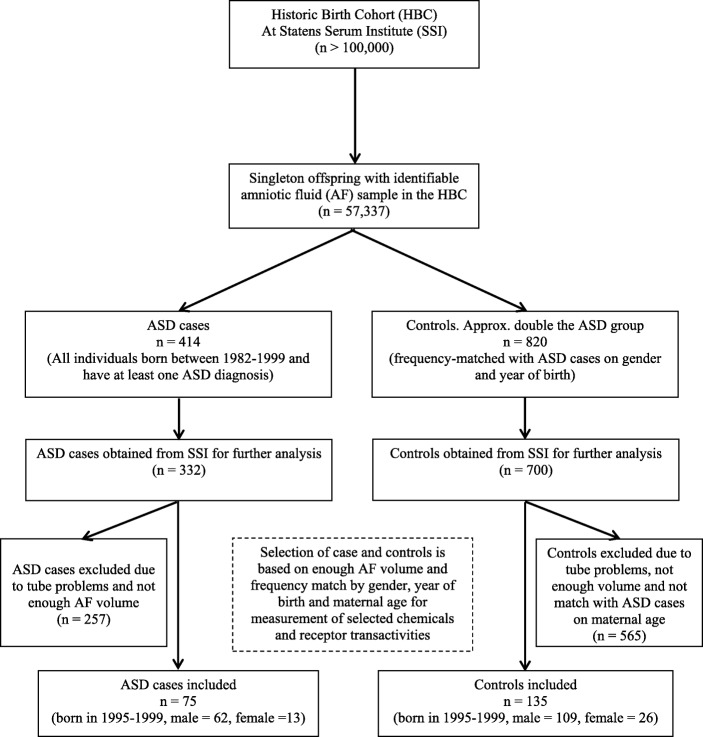
Flow chart of autism disorders (ASD) and controls selection process

Figure 1 presents the case and control selection process. Study subjects were initially selected from a population of singleton births between 1982 and 1999 with AF samples available as part of the Historic Birth Cohort (HBC) stored at the Statens Serum Institute (SSI) in Copenhagen, Denmark [47]. The HBC is based on a collection of antenatal biological samples obtained during screening/diagnostic procedures performed mainly in three Danish regions. The collection of samples goes from the late 1970s until 2004 and includes more than 100,000 samples of AF, bloodspots, and maternal serum samples (Fig. 1) [14, 48]. The AF samples from the HBC were centrifuged after routine screening or diagnostic amniocentesis, and samples were kept frozen at − 20 °C until further analyzed [49]. The Danish nation-wide health registers were employed to follow-up individuals in the HBC until 2009. All psychiatric diagnoses were identified utilizing the Danish Psychiatric Central Register (DPCR) which has high diagnosis validity of infantile autism diagnoses [50]. All singleton ASD cases born during 1982–1999 were identified according to the International Classification of Diseases (ICD)-8 codes 299.*xx* up to 1993 and ICD-10 codes DF84.*xx* since 1994. Furthermore, the Danish National Hospital Register (DNHR) primary diagnoses [51] were applied to complement diagnoses of congenital malformations and other psychiatric comorbidities. The birth record data of the study subjects were retrieved from the Danish medical birth registry [52]. The controls were non-ASD individuals randomly retrieved from the HBC and frequency-matched with cases on gender and year of birth [47].

The present study aims to examine whether EDCs in AF influence individuals diagnostics with ASD later in life using the case-control design. We first performed a pilot study on pooled AF to establish methods for the EDC-receptor function analyses and measurement of POPs such as PCBs, OCPs, and PFAS as well as elements including heavy metals. The pilot study showed that the levels of lipophilic POPs (PCBs, OCPs, dioxins, PBDEs) in AF samples were below the detection limits while PFAS, elements/metals, and AF-induced combined receptor transactivities could be detected in AF samples. Therefore, 1032 individual AF samples including 332 ASD cases and 700 controls were obtained from the SSI and stored at − 20 °C in Centre for Arctic Health & Molecular Epidemiology, Department of Public Health, Aarhus University, Denmark, for the determination of PFAS, elements, and the combined ex vivo receptor transactivities (ER, AR, AhR, and TH). Since many parameters had to be determined in each AF sample, the first selection of samples was based on available samples having adequate volume. AF samples of ASD cases and controls with adequate volume were thus selected and frequency-matched by gender and year of birth. Due to the possible influence of maternal age on ASD, the ASD cases and controls were further matched by maternal age with approximately 1:2 case-control match (Fig. 1).

Changes in concentrations of analytes over prolonged times are a known issue [53], and the way the samples were stored pre- and post-1993 was different. Baron-Cohen reported the evidence of evaporation and the concentrations of various analytes of pre-1993 samples were higher than those of post-1993 samples [14]. In addition, after 1993, the timing of amniocentesis in Denmark was standardized using ultrasound to mark gestational age and diagnostic information after 1993 became much more reliable by switching to ICD-10 [50, 54]. Therefore, the present study was restricted to individuals born between 1993 and 1999. However, several samples were stored in tubes with blue rubber caps containing cell toxic compounds affecting cell culture growth. Those samples were excluded from the present study. Finally, 75 ASD cases (62 boys, 13 girls) and 135 frequency-matched controls (109 boys, 26 girls) born during 1995–1999 with adequate AF volume were included in the study (Fig. 1).

### Perfluoroalkyl substances (PFAS) measurements

The concentrations of PFAS in AF were measured at the Institute for Environmental Science, Aarhus University, Denmark. The extraction method was based on ion pairing as described previously [55]. Six perfluorosulfonated acids (PFSA) and eight perfluorocarboxylated acids (PFCA) were determined by liquid chromatography-tandem mass spectrometry (LC-MS-MS) with electrospray ionization (ESI) [56]. The measured PFSA were perfluorooctane sulfonate (PFOS), perfluoroheptane sulfonate (PFHpS), perfluorobutane sulfonate (PFBS), perfluorohexane sulfonate (PFHxS), perfluorodecane sulfonate (PFDS), and perfluorooctane sulfonamide (PFOSA). The sum of PFOS, PFHpS, PFBS, PFHxS, PFDS, and PFOSA was given as ∑PFSA. The measured PFCA were perfluorooctanoic acid (PFOA), perfluorohexanoic acid (PFHxA), perfloroheptanoate (PFHpA), perfluorononanoic acid (PFNA), perfluorodecanoic acid (PFDA), perfluoroundecanoic acid (PFUnA), perfluorododecanoic acid (PFDoA), and perfluoro-n-pentanoic acid (PFPeA). The sum of PFOA, PFHxA, PFHpA, PFNA, PFDA, PFUnA, PFDoA, and PFPeA was given as ∑PFCA. Half of the limit of quantification values were used for concentrations below the limit of quantification (Additional file 1).

### Measurement of elements and metals

The concentrations of 59 elements and heavy metals in AF were determined using inductively coupled plasma mass spectrometry (ICP-MS) after dilution 20 times with 5% nitric acid at the Institute for Bioscience-Arctic Research Centre, Aarhus University, Denmark. The quality was ensured by participation in the Quality Assurance of Information in Marine Environmental monitoring (QUASIMEME) inter-laboratory comparison program [57]. The detection limits of measured elements were obtained as three times the standard deviation of blind (acid alone). Half of the detection limit values were used for concentrations below the detection limit (Additional file 2).

### Measurement of endogenous sex hormones

The endogenous sex hormone levels in AF were determined by solid phase fluoroimmunoassays. The DELFIA® Estradiol kit and Testosterone kit (PerkinElmer Life and Analytical Sciences, Wallac Oy, Turku, Finland) [58] were used to measure the levels of estrogen 17β-Estradiol (E2) and androgen testosterone, respectively, at the Centre of Arctic Health & Molecular Epidemiology, Department of Public Health, Aarhus University, Denmark. The DELFIA kits offer a faster method for measuring estradiol and testosterone with adequate sensitivity compared to radioimmunoassays [59]. The detection limit was 13.62 pg/mL and 115.4 pg/mL for E2 and testosterone, respectively. The intra-assay and inter-assay coefficient of variation was 10.0% and 9.7% for E2 and 5.7% and 8.4% for testosterone, respectively.

### Measurement of receptor transactivities induced by AF

To evaluate the potential of the compounds/molecules in AF to interfere with the estrogen receptor (ER), androgen receptor (AR), and aryl hydrocarbon receptor (AhR) transactivation, the luciferase reporter gene transactivities (tact) of these receptors (ER-tact, AR-tact, and AhR-tact) were determined. For thyroid hormone (TH)-related action, the proliferative effect (TH-PE) of AF was determined using a TH-dependent GH3 cell proliferation assay (T-screen assay) at the Centre for Arctic Health & Molecular Epidemiology, Department of Public Health, Aarhus University, Denmark. The established luciferase reporter gene bioassays [20, 27, 60] and the T-screen assay [21] have been described earlier in detail.

The AF-induced ER-tact was determined by direct exposure of the stably transfected MVLN cells to 6.25 μl AF dissolved in 100 μl of culture medium (CD-FCS DMEM). In parallel, an eight-point E2 series (3.1–300 pM) was included as a positive control and used for estradiol equivalent (ER-EEQ) calculation.

The AF-induced AR-tact was determined by direct exposure of transiently transfected CHO-K1 cells to 5 μl AF dissolved in 100 μl of culture medium (DMEM/F12 + 10% CD-FCS). In parallel, an eight-point dihydrotestosterone (DHT) series (2–200 pM) was included as a positive control and used for calculation of the androgen equivalent (AR-AEQ).

The TH-disrupting potential of AF (TH-PE) was analyzed in the T-screen assay by exposing the GH3 cells to 14 μl of AF in 200 μl thyroid hormone-depleted CD-FCS DMEM. In parallel, an eight-point triiodothyronine (T3) series (10–10,000 pM) was included as a positive control and for calculation of the thyroid equivalent (T3-ThEQ).

We measured the AhR transactivity (AhR-tact) of AF by exposure of Hepa1.12cR cells to an AF extract. The AF extracts were prepared as follows: 500 μl AF was extracted on a solid phase extraction (SPE) column (Discovery C18, Sigma-Aldrich), eluted with hexane and evaporated to near dryness. The SPE extract of AF was dissolved in 1 μl DMSO, and 400 μl of culture medium (alpha MEM) was added. The cells were exposed to 100 μl/well of this solution of AF extract, and the AhR-tact was determined. As procedural blank, 500 μl distilled water was extracted, processed, and analyzed in the same way as the AF samples. In parallel, a nine-point 2, 3, 7, 8-tetrachlorodibenzo-*p*-dioxin (TCDD, potent AhR ligand) series (2–5000 pM) was included as a positive control and used for calculation of the AhR-TCDD equivalent (AhR-TEQ).

### Calculation of receptor ligand equivalent (EQ)

The combined effect on the hormone receptors induced by the endogenous hormones and EDCs present in AF was calculated as the corresponding receptor ligand equivalent (EQ). Table 1 shows an explanation of measured receptor transactivities induced by AF and calculation of EQ. Briefly, the receptor ligand EQ was calculated by interpolation of the measured transactivity value of ER-tact, AR-tact, and TH-PE on the corresponding ligand dose-response four-parameter sigmoidal Hill curve and expressed as ER-EEQ (pg E2/mL AF), AR-AEQ (pg DHT/mL AF), and T3-ThEQ (ng T3/mL AF), respectively. The integrated xenoestrogenic effect induced by EDCs alone was estimated by subtracting concentration of endogenous E2 from ER-EEQ and expressed as EDC-EEQ (pg E2/mL AF). For those ER-EEQ values less than the levels of E2, half of E2 detect limit was given as their EDC-EEQ values. We did not measure the DHT level but the testosterone level. The literature reported that almost 10% of the testosterone is converted to DHT [61]. In our lab, we observed that the potential of testosterone activating AR is 10 times less than DHT; this is similar to a previous study [62]. Thus, we estimated the level of endogenous DHT to be 10% of measured testosterone and subsequently the EDC-induced xenoandrogenic effect was obtained by subtracting the estimated endogenous DHT level from AR-AEQ and expressed as EDC-AEQ (pg DHT/mL AF) (Table 1). For those AR-AEQ values lower than levels of DHT, 10% of half testosterone detect limit was given as their EDC-AEQ values. To explore whether the endogenous sex hormones and/or EDCs influence the balance between androgenic effect and estrogenic effect, the ratio of AR-AEQ to ER-EEQ (AR-AEQ/ER-EEQ) and ratio of EDC-AEQ to EDC-EEQ (EDC-AEQ/EDC-EEQ) were further calculated. AR-AEQ/ER-EEQ represents the proportion of total combined androgenic effect to the total combined estrogenic effect. EDC-AEQ/EDC-EEQ reflects the proportion of EDC-induced androgenic effect to EDC-induced estrogenic effect.

**Table 1:** Explanation of measured receptor transactivities induced by amniotic fluid (AF) and receptor ligand equivalent calculation

Abbreviations	Explanation	Example of calculation
ER-tact	Luciferase reporter gene transactivity of estrogen receptor (ER)	
ER-EEQ	The combined effect on ER induced by the combination of endogenous estrogen and xeno-estrogens (EDCs) given as estradiol equivalent	• ER-tact of AF: 2.51 • Interpolation of ER-tact onto E2 dose-response curve • ER-EEQ = 247.60 pg E2/ml AF
EDC-EEQ	The integrated estrogenic effect induced by xeno-estrogens (EDCs) alone given as estradiol equivalent	• Endogenous E2: 230.23 pg/ml • (ER-EEQ) − E2 = 247.60 − 230.32 = 17.28 E2 pg/ml AF
AR-tact	Luciferase reporter gene transactivity of androgen receptor (AR)	
AR-AEQ	The combined effect on AR induced by the combination of endogenous androgens and xeno-androgens (EDCs) given as androgen equivalent	• AR-tact of AF: 3.30 • Interpolation of AR-tact onto DHT dose-response curve • AR-AEQ = 63.50 pg DHT/ml AF
EDC-AEQ	The integrated androgenic effect induced by xeno-androgens (EDCs) alone given as androgen equivalent	• Testosterone: 470.12 pg/ml AF • DHT: estimated DHT = 10% × 470.12 = 47.01 pg/ml AF • (AR-AEQ) − DHT = 63.50 − 47.01 = 16.49 pg DHT/ml AF
AR-AEQ/ER-EEQ	The proportion of total combined androgenic effect to the total combined estrogenic effect	• AR-AEQ = 63.50 pg DHT/mlAF • ER-EEQ = 247.60 pg E2/mlAF • AR-AEQ/ER-EEQ = 63.50/247.60 = 0.256
EDC-AEQ/EDC-EEQ	The proportion of EDC-induced androgenic effect to EDC-induced estrogenic effect	• EDC-AEQ = 16.49 DHT/mlAF • EDC-EEQ = 17.28 E2/mlAF • EDC-AEQ/EDC-EEQ = 16.49/17.28 = 0.954
TH-PE	Thyroid hormone-related proliferative effect	
T3-ThEQ	The combined proliferation effect (PE) of endogenous thyroid hormone and xeno-thyroid hormone-like compounds (EDCs) given as thyroid equivalent	• TH-PE of AF: 4.56 • Interpolation of TH-PE onto T3 dose-response curve • T3-ThEQ = 3.53 ng T3/ml AF
AhR-tact	Luciferase reporter gene transactivity of aryl hydrocarbon receptor (AhR)	
AhR-TEQ	The combined effect on AhR of dioxin-like compounds given as TCDD equivalent	• AhR-tact of AF extraction: 5.67 • AhR-tact of blank: 4.75 • Interpolation of AhR-tact onto TCDD dose-response curve = AhR-TEQ • AhR-TEQ of AF extraction = 1.46 pg TCDD/ml, • AhR-TEQ of blank = 1.22 pg TCDD/ml • Final AhR-TEQ = 1.46 − 1.22 = 0.24 pg TCDD/ml AF

*AF* amniotic fluid, *AEQ* androgen equivalent, *AR* androgen receptor, *AhR* aryl hydrocarbon receptor, *DHT* dihydrotestosterone, *EDCs* endocrine disrupting compounds, *EEQ* estradiol equivalent, *ER* estrogen receptor, *E2* 17 β-Estradiol, *PE* proliferation effect, *TCDD* 2, 3, 7, 8-tetrachlorodibenzo-p-dioxin, *TEQ* TCDD equivalent, *T3* triiodothyronine, *ThEQ* thyroid equivalent

The TCDD equivalent (AhR-TEQ) values of the AF extracts and procedural blank were estimated by interpolation of their AhR-tact values on the TCDD dose-response three-parameter sigmoidal Hill curve. The final net AhR–TEQ of AF (pg TCDD/mL AF) was obtained by subtracting the procedure blank TEQ value (Table 1).

### Statistical analysis

Only the chemicals of interest and receptor transactivities that were detectable in at least 10% of the AF samples were included in the statistical analyses. Because not all biomarkers were detected in all AF samples and data were skewed, we compared the differences of exposure biomarkers between the ASD cases and controls using the non-parametric Mann-Whitney test.

We used Spearman’s correlation analysis to analyze the correlation of PFAS, elements, receptor transactivities, and potential confounders.

To analyze the association of prenatal EDC exposure and ASD risk, unconditional logistical regression analysis was used to estimate the odds ratio (OR) and 95% confidence interval (95% CI). The levels of PFAS, elements, and receptor transactivities were included as continuous variables into the model with and without potential confounders.

The change in estimate principle [63] was used to identify the confounders using a cutoff of 5%. Potential confounders available for this analysis included birth year and gender of child, maternal age at child birth, paternal age at child birth, birth weight, gestational age at birth, gestational week at sampling, Apgar score, and parity based on a priori consideration of the research design and ASD risk factors [64–66]. Given that ASD risk is also driven by genetic factors, we further adjusted for congenital malformation.

Twenty-nine individual exposure variables were detected in more than 10% of samples, and multiple comparisons were performed. We controlled for multiple comparisons using false discovery rate (FDR; *q*_value_) correction as suggested by Benjamini-Hochberg [67, 68]. Since the present study is the first study to explore the associations of autism risk with chemicals and AF-induced receptor transactivity, a false discovery rate of 0.25 (FDR *q*_value_ < 0.25) was used as a significant level for the multiple comparison correction [69].

ASD possesses gender difference with male preponderance [70], and ASD is associated with a higher rate of congenital malformation [71]. In addition, we suspected possible modification effect of gender. We therefore further performed the sensitivity analysis by stratifying on children’s gender and excluding congenital malformation diagnoses.

#### Principal component analysis

The use of a large number of individual parameters would create a certain number of false positive results when performing multiple comparisons. Principal component analysis (PCA) is a powerful multivariate analytical tool and is applied to reduce a set of original variables and to extract a smaller number of principal components. Using PCA for data transformation is a common ad hoc tool to deal with multicollinearity in multiple regression models because the correlation between related compounds such as PFAS congeners exists and this multicollinearity biases the estimation of individual regression coefficients to cause misleading interpretation of the effects of individual predictor variables [72, 73]. PCA reduces the false positives by reducing the number of variables [74]. Besides, people are exposed to the mixture of different substances simultaneously, it is important to assess the effect of actual effect of mixture and PCA might provide the possibility to address this issue. Therefore, we further used PCA to identify potential underlying components of levels of PFAS, metals, and receptor transactivities in AF. By PCA, the correlated variables are grouped together. The coefficients defining these linear combinations, termed as “factor loadings,” are the correlations of each input variables with the component. The biomarkers which were detectable in more than 10% of all AF samples were input into the model to extract principal components (PCs). The number of PCs was extracted based on the eigenvalues > 1 and Varimax rotation [75]. The Kaiser-Mayer-Olkin (KMO) test (> 0.500) and Bartlett Test of Sphericity (*p* < 0.001) were used for testing the suitability of analysis. All factors loading > |0.3| were used to identify the variables comprising a PC.

The difference of identified PCs between cases and controls was analyzed by Mann-Whitney test. The association of identified PCs and ASD risk was also analyzed by logistic regression model with and without adjustment of potential confounders. FDR correction and sensitivity analysis were also performed for the identified principle components.

All statistical analyses were performed using SPSS version 24.0 (SPSS Inc. Chicago, IL, USA) conducted at the Centre of Arctic Health & Molecular Epidemiology, Department of Public Health, Aarhus University, Denmark. The statistical significance level was set to *p* ≤ 0.05. For multiple comparison correction, the statistical level was set to FDR *q*_value_ < 0.25.

## Results

### Characteristics of the study population

Table 2 shows the descriptive characteristics of the study population. Most children were male and born at term. The majority of AF samples were obtained in gestational week 15. More than 62% of mothers were carrying at least two children. The demographics of cases and controls were similar. The percentages of Apgar scores > 7 were above 98% both in case and control groups, indicating that almost all participants in this study were classified as healthy at birth. ASD cases were significantly more co-diagnosed with congenital malformation (29.30% vs. 8.10%, *p* < 0.0001).

**Table 2:** Characteristics of the study population

Characteristic	ASD cases (*n* = 75)	Controls (*n* = 135)	*p* value
Mother’s age at delivery (median, min-max, year)	34 (17–41)	35 (21–44)	0.218
Father’s age at delivery (median, min-max, year)	35 (19–53)	35 (20–58)	0.362
Gestational week at sampling (median, min-max)	15 (11–20)	15 (10–29)	0.426
Parity (*n*, %)			0.797
1st child	28 (37.30%)	48 (35.6%)	
> 2nd child	47 (62.70%)	87 (64.4%)	
Gestational age at birth (*n*, %)			0.424
Preterm (< 260 days)	7 (9.30%)	15 (11.20%)	
Term (260 days–294 days)	67 (89.30%)	113 (84.30%)	
Post-term (> 295 days)	1 (1.30%)	6 (4.50%)	
Gender of the children (*n*, %)			0.731
Female	13 (17.30%)	26 (19.30%)	
Male	62 (82.70%)	109 (80.70%)	
Birth weight of the children (*n*, %)			0.521
< 2500 g	3 (4.10%)	10 (7.50%)	
2500 g–4000 g	57 (77.00%)	95 (70.90%)	
> 4000 g	14 (18.90%)	29 (21.60%)	
Apgar score of the children (*n*, %)			0.952
> 7	70 (98.60%)	130 (98.50%)	
< 7	1 (1.40%)	2 (1.50%)	
Congenital malformation of the children (*n*, %)			*< 0.0001*
No	53 (70.70%)	124 (91.90%)	
Yes	22 (29.30%)	11 (8.10%)	

Student *t* test and chi-square test were used to compare the difference between cases and controls. Italicized values indicate statistically significant

### Levels of perfluoroalkyl substances (PFAS)

PFOS, PFOSA, PFHxS, PFOA, and PFDoA were detectable in 46.6%, 35.2%, 4.50%, 81.8%, and 1.10% of the AF samples, respectively; the remaining PFAS congeners were under the limit of quantification (Additional file 1). For the control group, no significant correlations between PFAS congeners and potential confounders were observed, and PFOS was significantly correlated to PFOA (*r* = 0.620, *p* < 0.0001), PFHxS (*r* = 0.330, *p* = 0.02), and borderline to PFOSA (*r* = 0.300, *p* = 0.056) (data not shown).

Cases significantly differed from controls with lower levels of PFOS (*p* = 0.015) and ∑PFSA (*p* = 0.011) as well as all the tested PFAS (∑PFSA+∑PFCA) (*p* = 0.021) (Table 3). After false discovery rate correction, the significances persisted (FDR *q*_value_ = 0.189, Table 3).

**Table 3:** Levels of perfluoroalkylated substances and elements in amniotic fluid

	ASD cases			Control				
	*n*	Median	Min-max	*n*	Median	Min-max	*p*	FDR (*q*_value_)
PFAS (ng/mL)								
PFOS	37	0.61	0.61–2.98	51	1.44	0.61–4.22	*0.015*	*0.189*
PFOSA	37	0.60	0.60–13.30	51	0.60	0.60–19.00	0.152	0.638
PFOA	37	0.29	0.10–0.78	51	0.32	0.10–1.86	0.516	0.764
∑PFSA	37	1.56	1.56–15.40	51	2.60	1.56–21.70	*0.011*	*0.189*
∑PFCA	37	1.54	1.35–2.03	51	1.57	1.35–3.11	0.489	0.764
∑PFSA+∑PFCA	37	3.26	2.91–17.37	51	4.23	2.91–23.26	*0.021*	*0.189*
Elements (μg/L)								
Fe	37	259.00	16.00–778.0	51	270.00	128.00–2575	0.571	0.764
Cu	37	89.15	45.62–206.10	51	100.40	53.53–223.10	0.189	0.638
Zn	37	97.90	34.69–754.5	51	102.00	30.90–1216	0.557	0.764
Se	37	4.43	2.250–7.130	51	4.55	2.980–7.27	0.758	0.861
I	37	1.88	1.88–94.68	51	1.88	1.88–195.40	0.765	0.861
Cr	37	0.75	0.16–3.85	51	0.73	0.31–2.21	0.365	0.704
Mn	37	0.65	0.65–25.89	51	0.65	0.65–5.26	0.826	0.892
As	37	1.33	1.33–6.19	51	1.33	1.33–4.96	0.328	0.704
Cd	37	0.02	0.02–1.92	51	0.02	0.02–0.14	0.648	0.795
Pb	37	0.22	0.22–2.27	51	0.22	0.22–5.30	0.526	0.764

*∑PFSA* perfluorosulfonated acids which was the sum of PFOS, PFHpS, PFBS, PFHxS, PFDS, and PFOSA, *∑PFCA* perfluorocarboxylated acids which was the sum of PFOA, PFHxA, PFHpA, PFNA, PFDA, PFUnA, PFDoA, and PFPeA, *FDR (q*_*value*_*)* false discovery rate. The difference between cases and controls was compared using non-parametric Mann-Whitney test. Italicized values indicate statistically significant (*p* < 0.05 and FDR *q*_value_ < 0.25)

### Levels of elements and heavy metals

Among the measured 59 elements and metals, 16 elements including iron (Fe), copper (Cu), zinc (Zn) and selenium (Se) were detectable in all AF samples, while 8 elements, including mercury (Hg) and silver (Ag), were not detectable in any AF samples. The metals of interest such as chromium (Cr), lead (Pb), arsenic (As), manganese (Mn), and cadmium (Cd) were detected in 98.9%, 34.1%, 22.7%, 18.2%, and 12.5% of the AF samples, respectively (Additional file 2).

Similar levels of elements and heavy metals between ASD cases and controls were observed (Table 3).

### Levels of endogenous sex hormones and the combined effect of endogenous hormones and EDCs on the receptor transactivation

For the endogenous sex hormone levels, no significant difference between ASD cases and controls was observed. The ratio of testosterone to E2 was slightly higher in ASD cases compared to controls (Table 4).

**Table 4:** Levels of hormones and receptor transactivities in amniotic fluid

Parameter	ASD case			Control				
	*n*	Median	Min-Max	*N*	Median	Min-Max	*p*	*FDR* (*q*_value_)
E2 (pg/mL)	68	287.4	78.72–2778	128	311.9	55.02–1005	0.337	0.704
Testosterone (pg/mL)	67	383.6	47.30–1393	130	366.3	80.47–1090	0.173	0.638
Testosterone/E2 ratio	66	1.49	0.21–7.41	128	1.25	0.14–4.310	0.222	0.666
ER-EEQ (pg E2/mL)	75	470.9	204.1–1308	135	545.8	149.0–1308	0.329	0.704
EDC-EEQ (pg E2/mL)	68	168.5	0.28–1226	128	214.4	2.72–1165	0.162	0.638
AR-AEQ (pg DHT/mL)	75	62.60	23.90–137.3	135	62.50	16.90–290.4	0.910	0.910
EDC-AEQ (pg DHT/mL)	67	22.56	1.000–100.4	130	28.45	1.34–274.0	0.167	0.638
AR-AEQ/ER-EEQ	75	0.13	0.02–0.48	135	0.11	0.02–0.98	0.343	0.704
EDC-AEQ/EDCs-EEQ	61	0.18	0.01–59.10	117	0.086	0.003–26.21	0.515	0.764
T3-ThEQ (ng T3/mL)	74	2.36	0.10–17.14	133	2.41	0–6.60	0.594	0.764
AhR-TEQ (pg TCDD/mL)	37	0.24	0–1.310	51	0.15	0–2.10	0.907	0.910

*E2* 17β-estradiol, *ER-EEQ* the combined effect on ER of endogenous estrogen and xeno-estrogens (EDCs) given as estradiol equivalent, *EDCs-EEQ* the integrated estrogenic effect induced only by xeno-estrogens (EDCs) alone given as estradiol equivalent, *DHT* dihydrotestosterone, *AR-AEQ* the combined effect on AR of endogenous androgens and xeno-androgens (EDCs) given as androgen equivalent, *EDC-AEQ* the integrated androgenic effect induced only by xeno-androgens (EDCs) alone given as androgen equivalent, *T3* L-3,5,3′-triiiodothyronine, *T3-ThEQ* the combined proliferation effect (PE) of endogenous thyroid and xeno-thyroid hormone-like compounds (EDCs) given as thyroid equivalent, *AhR-TEQ* the combined effect on AhR of dioxin-like compounds given as TCDD equivalent, *FDR (q*_*value*_*)* false discovery rate. See the “Methods” section and Table 1 for the different calculations. The difference between cases and controls was compared using non-parametric Mann-Whitney test

The effect of AF samples on the receptor transactivation is given as the corresponding receptor ligand equivalent (for detailed explanation see Table 1). We observed that the combined effect of endogenous estrogen and EDCs on ER (ER-EEQ) and EDC-induced xenoestrogenic effect (EDC-EEQ) was slightly lower in cases, but the difference was not significant (Table 4). We observed no significant differences between the ASD cases and controls for the combined androgenic effect (AR-AEQ) and neither for TH-like activity (T3-ThEQ) and dioxin-like activity (AhR-TEQ) (Table 4).

### Association of ASD risk and levels of chemicals

As given in Table 5, for the crude and adjusted data, we observed an inverse association between PFOS level in AF and ASD risk (adjusted OR 0.410, 95% CI 0.140–0.967, *p* = 0.042) (Table 5). However, after false discovery rate correction, the significance of PFOS disappeared (FDR *q*_value_ = 0.437, Table 5).

**Table 5:** Odds ratio and 95% confidence intervals for ASD according to PFAS in amniotic fluid

	Crude			Adjusted^a^			
	*n* (cases/controls)	OR (95% CI)	*p*	*n* (cases/controls)	OR (95% CI)	*p*	FDR (*q*_value_)
PFOS (ng/mL)	37/51	*0.460 (0.230, 0.921)*	*0.028*	37/50	*0.410 (0.174, 0.967)*	*0.042*	0.437
PFOSA (ng/mL)	37/51	0.913 (0.797, 1.046)	0.189	37/50	0.918 (0.789, 1.069)	0.272	0.565
PFOA (ng/mL)	37/51	0.335 (0.044, 2.559)	0.292	37/50	0.164 (0.013, 2.126)	0.167	0.437
∑PFSA (ng/mL)	37/51	0.894 (0.777, 1.028)	0.115	37/50	0.900 (0.771, 1.049)	0.178	0.437
∑PFCA (ng/mL)	37/51	0.299 (0.041, 2.160)	0.232	37/50	0.150 (0.013, 1.760)	0.131	0.437
∑PFSA+∑PFCA (ng/mL)	37/51	0.894 (0.779, 1.025)	0.107	37/50	0.896 (0.770, 1.044)	0.158	0.437

*FDR (q*_*value*_*)* false discovery rate. Italicized values indicate statistically significant (*p* < 0.05 and FDR *q*_value_ < 0.25)

^a^Adjusted for children’s birth year, children’s sex, mother age at delivery, father age at child birth, birth weight, gestational week at sampling, gestational age at birth, Apgar score, parity, and congenital malformation

We observed no clear association for nutrient elements such as Fe, Cu, Zn, Se, and I with ASD risk (Table 6). However, although no significant association between toxic metals and ASD risk was observed, we noticed that adjusted ORs were 1.496 (95% CI 0.924, 2.424; *p* = 0.101) for As and 1.300 (95% CI 0.657, 2.575; *p* = 0.451) for Pb, respectively (Table 6).

**Table 6:** Odds ratio and 95% confidence intervals for ASD according to elements in amniotic fluid

	Crude			Adjusted^a^			
	*n* (cases/controls)	OR (95% CI)	*p*	*n* (cases/controls)	OR (95% CI)	*p*	FDR (*q*_value_)
Fe (μg/L)	37/51	0.999 (0.996, 1.001)	0.255	37/50	0.999 (0.997, 1.001)	0.449	0.677
Cu (μg/L)	37/51	0.991 (0.978, 1.005)	0.211	37/50	0.997 (0.981, 1.012)	0.685	0.771
Zn (μg/L)	37/51	0.999 (0.996, 1.002)	0.673	37/50	0.999 (0.995, 1.002)	0.491	0.698
Se (μg/L)	37/51	0.977 (0.649, 1.469)	0.910	37/50	1.058 (0.644, 1.740)	0.823	0.823
I (μg/L)	37/51	0.996 (0.978, 1.014)	0.674	37/50	0.981 (0.959, 1.003)	0.087	0.437
Cr (μg/L)	37/51	1.019 (0.403, 2.576)	0.968	37/50	0.377 (0.051, 2.764)	0.337	0.650
Mn (μg/L)	37/51	1.106 (0.878, 1.394)	0.393	37/50	1.065 (0.841, 1.347)	0.602	0.771
As (μg/L)	37/51	1.257 (0.856, 1.846)	0.244	37/50	1.496 (0.924, 2.424)	0.101	0.437
Cd (μg/L)	37/51	4.418 (0.099, 197)	0.444	37/50	5.935 (0.101, 349.3)	0.392	0.662
Pb (μg/L)	37/51	1.086 (0.615, 1.918)	0.776	37/50	1.300 (0.657, 2.575)	0.451	0.677

*FDR (q*_*value*_*)* false discovery rate

^a^Adjusted for children’s birth year, children’s sex, mother age at delivery, father age at child birth, birth weight, gestational age at birth, gestational week at sampling, Apgar score, parity, and congenital malformation

After stratifying by the children’s gender and removing cases with congenital malformations, the results regarding PFAS, metals/elements, and ASD risk were not changed (Additional files 3 and 4).

### Association of ASD risk with the levels of sex hormones and the combined AF-induced receptor effect biomarkers

The logistic regression analyses showed a weak association between AF testosterone level and ASD risk (adjusted OR 1.002; 95% CI 1.000, 1.004; *p* = 0.050) (Table 7). However, after false discovery rate correction, this significant association did not persist (FDR *q*_value_ = 0.437, Table 7).

**Table 7:** Odds ratio and 95% confidence intervals for ASD according to hormone level and receptor transactivity induced by amniotic fluid

	Crude			Adjusted^a^			
	*n* (cases/controls)	OR (95% CI)	*p*	*n* (cases/controls)	OR (95% CI)	*p*	FDR (*q*_value_)
E2 (pg/mL)	68/128	1.000 (0.999, 1.001)	0.629	65/125	1.000 (0.999, 1.001)	0.669	0.771
Testosterone (pg/mL)	67/130	*1.001 (1.000, 1.003)*	*0.050*	64/127	*1.002 (1.000, 1.004)*	*0.050*	0.437
Testosterone/E2	66/128	1.257 (0.950, 1.664)	0.110	63/125	1.350 (0.935, 1.949)	0.110	0.437
ER-EEQ (pg E2/mL)	75/135	1.000 (0.999, 1.000)	0.358	71/132	1.000 (0.999, 1.000)	0.222	0.500
EDCs-EEQ (pg E2/mL)	68/128	0.999 (0.998, 1.000)	0.112	65/125	0.999 (0.998, 1.000)	0.089	0.437
AR-AEQ (pg DHT/mL)	75/135	0.995 (0.985, 1.006)	0.389	71/132	0.997 (0.985, 1.010)	0.650	0.771
EDCs-AEQ (pg DHT/mL)	67/130	0.989 (0.976, 1.002)	0.109	64/127	0.989 (0.974, 1.005)	0.169	0.437
AR-AEQ/ER-EEQ	75/135	1.269 (0.104, 15.479)	0.852	71/132	2.176 (0.115, 41.15)	0.604	0.771
EDCs-AEQ/EDCs-EEQ	66/128	1.022 (0.794, 1.552)	0.423	63/125	1.032 (0.974, 1.092)	0.285	0.662
T3-ThEQ (ng T3/mL)	74/133	1.094 (0.874, 1.369)	0.434	71/130	1.036 (0.806, 1.332)	0.784	0.822
AhR-TEQ (pgTCDD/mL)	37/51	0.786 (0.238, 2.589)	0.692	36/50	0.792 (0.192, 3.270)	0.747	0.822

*E2* 17β-Estradiol, *ER-EEQ* the combined effect on ER of endogenous estrogen and xeno-estrogens (EDCs) given as estradiol equivalent, *EDC-EEQ* the integrated estrogenic effect induced only by xeno-estrogens (EDCs) alone given as estradiol equivalent, *DHT* dihydrotestosterone, *AR-AEQ* the combined effect on AR of endogenous androgens and xeno-androgens (EDCs) given as androgen equivalent, *EDC-AEQ* the integrated androgenic effect induced only by xeno-androgens (EDCs) alone given as androgen equivalent, *T3* L-3,5,3′-triiiodothyronine, *T3-ThEQ* the combined proliferation effect (PE) of endogenous thyroid and xeno-thyroid hormone-like compounds (EDCs) given as thyroid equivalent, *AhR-TEQ* the combined effect on AhR of dioxin-like compounds given as TCDD equivalent, *FDR (q*_*value*_*)* false discovery rate. Italicized values indicate statistically significant (*p* < 0.05 and FDR *q*_value_ < 0.25)

^a^Adjusted for children’s birth year, children’s sex, mother age at delivery, father age at child birth, birth weight, gestational age at birth, gestational week at sampling, Apgar score, parity, and congenital malformation

We found no significant association between ASD risk and AF-induced receptor transactivities. We noticed, however, that the adjusted OR for the ratio of AR-AEQ/ER-EEQ was 2.176 (95% CI 0.115, 41.15). We observed no obvious associations between ASD risk and dioxin-like activity (AhR-TEQ) and TH-like activity (Table 7). Stratifying by children’s gender and removing the congenital malformations did not alter the results (Additional files 4 and 5).

### Correlations between PFAS levels and combined AF-induced receptor effect biomarkers

In general, PFAS positively correlated with the ER-EEQ and EDC-EEQ being significant for PFOS, PFOSA, ΣPFSA, and all tested PFAS (∑PFSA+∑PFCA). In contrast, PFAS inversely correlated with AR-AEQ as well as EDC-AEQ, being significant for cases (Table 8).

**Table 8:** The correlation among PFAS levels in amniotic fluid and receptor transactivity induced by AF

	ER-EEQ			EDC-EEQ			AR-AEQ			EDC-AEQ			T3-ThEQ			AhR-TEQ		
	*n*	*r*_s_	*p*	*n*	*r*_s_	*p*	*n*	*r*_s_	*p*	*n*	*r*_s_	*p*	*n*	*r*_s_	*p*	*n*	*r*_s_	*p*
ASD cases																		
PFOS	37	*0.522*	*0.001*	30	0.246	0.189	37	− 0.258	0.124	30	*− 0.466*	*0.009*	34	0.145	0.398	37	0.097	0.569
PFOSA	37	0.237	0.158	30	0.199	0.292	37	*− 0.391*	*0.017*	30	− 0.163	0.390	36	− 0.038	0.825	37	0.140	0.410
PFOA	37	0.225	0.180	30	0.317	0.087	37	0.007	0.968	30	*− 0.399*	*0.029*	34	0.039	0.822	37	0.125	0.462
∑PFSA	37	*0.332*	*0.045*	30	0.158	0.403	37	− 0.305	0.066	30	− 0.310	0.095	34	− 0.026	0.882	37	0.100	0.555
∑PFCA	37	0.225	0.180	30	0.317	0.087	37	0.007	0.968	30	*− 0.399*	*0.029*	34	0.039	0.822	37	0.125	0.462
∑PFSA+∑PFCA	37	*0.343*	*0.038*	30	0.330	0.075	37	− 0.194	0.249	30	− 0.318	0.087	34	− 0.005	0.978	37	0.138	0.417
Controls																		
PFOS	51	0.156	0.276	44	0.273	0.073	51	− 0.082	0.565	46	− 0.010	0.947	48	− 0.004	0.979	51	− 0.065	0.649
PFOSA	51	0.220	0.121	44	*0.356*	*0.018*	51	− 0.160	0.263	46	− 0.108	0.476	49	− 0.221	0.127	51	− 0.273	0.1053
PFOA	51	0.104	0.469	44	0.097	0.530	51	− 0.002	0.989	46	0.048	0.751	48	0.209	0.150	51	0.148	0.300
∑PFSA	51	*0.321*	*0.021*	44	*0.442*	*0.003*	51	− 0.160	0.263	46	− 0.071	0.641	48	− 0.186	0.202	51	− 0.253	0.074
∑PFCA	51	0.100	0.487	44	0.093	0.548	51	− 0.005	0.974	46	0.051	0.736	48	0.203	0.161	51	0.142	0.322
∑PFSA+∑PFCA	51	*0.332*	*0.017*	44	*0.438*	*0.003*	51	− 0.146	0.305	46	− 0.047	0.757	48	− 0.148	0.309	51	− 0.217	0.125
All																		
PFOS	88	*0.305*	*0.004*	74	*0.251*	*0.031*	88	− 0.182	0.090	76	− 0.085	0.465	85	0.18	0.871	88	0.015	0.891
PFOSA	88	*0.224*	*0.036*	74	*0.304*	*0.009*	88	*− 0.258*	*0.015*	76	− 0.114	0.328	85	− 0.141	0.197	88	− 0.120	0.267
PFOA	88	0.194	0.070	74	0.212	0.070	88	− 0.031	0.777	76	− 0.127	0.274	82	0.117	0.285	88	0.145	0.178
∑PFSA	88	*0.342*	*0.001*	74	*0.354*	*0.02*	88	*− 0.259*	*0.015*	76	− 0.114	0.328	82	− 0.119	0.278	88	− 0.106	0.326
∑PFCA	88	0.191	0.074	74	0.210	0.073	88	− 0.033	0.758	76	− 0.123	0.288	82	0.115	0.294	88	0.142	0.187
∑PFSA+∑PFCA	88	*0.361*	*0.001*	74	*0.398*	*< 0.001*	88	*− 0.218*	*0.042*	76	− 0.103	0.374	82	− 0.088	0.423	88	− 0.065	0.545

*ER-EEQ* the combined effect on ER of endogenous estrogen and xeno-estrogens (EDCs) given as estradiol equivalent, *EDC-EEQ* the integrated estrogenic effect induced only by xeno-estrogens (EDCs) alone given as estradiol equivalent, *DHT* dihydrotestosterone, *AR-AEQ* the combined effect on AR of endogenous androgens and xeno-androgens (EDCs) given as androgen equivalent, *EDC-AEQ* the integrated androgenic effect induced only by xeno-androgens (EDCs) alone given as androgen equivalent, *T3* L-3,5,3′-triiiodothyronine, *T3-ThEQ* the combined proliferation effect (PE) of endogenous thyroid and xeno-thyroid hormone-like compounds (EDCs) given as thyroid equivalent, *AhR-TEQ* the combined effect on AhR of dioxin-like compounds given as TCDD equivalent, *r*_*s*_, Spearman’s rho correlation coefficient. Italicized values indicate statistically significant (*p* < 0.05)

### Principal component analysis

**Fig. 2:**
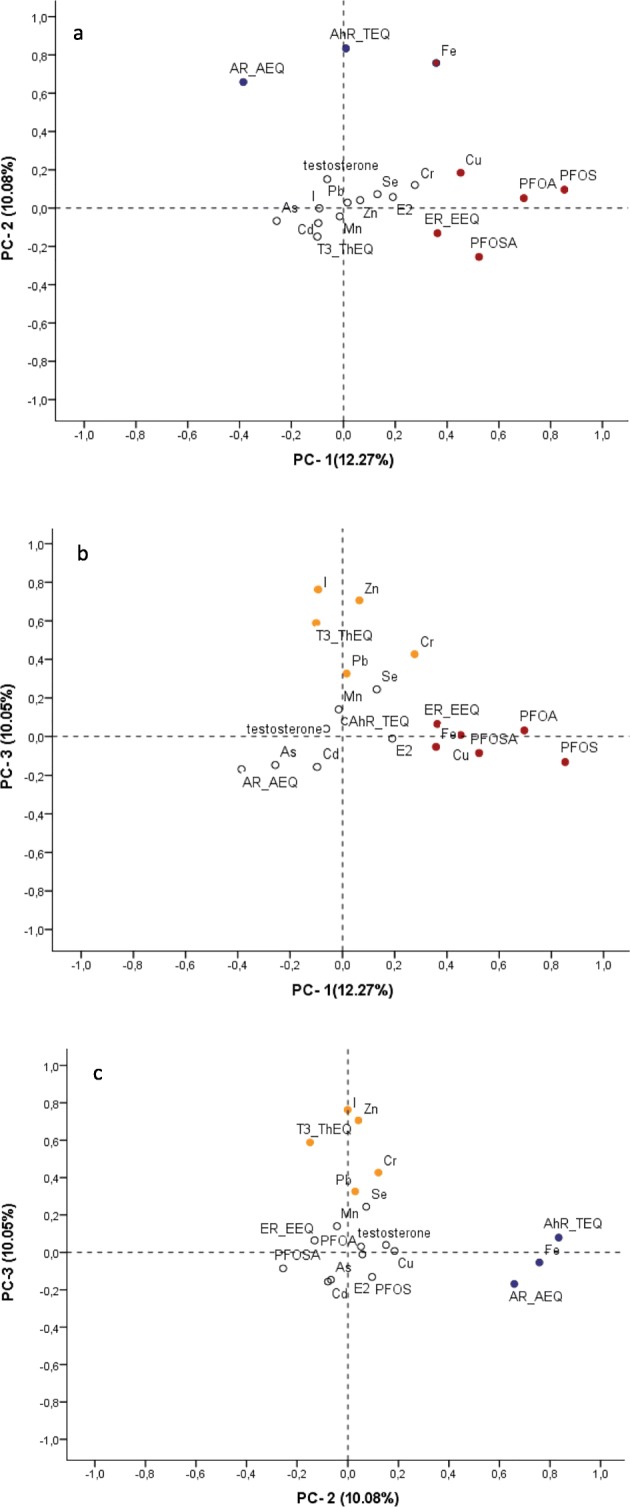
Plots of rotated factor loading of the three largest principal components (PCs) identified by principal component analysis (PCA). The percentage of variation (%) explained by each PC variable is presented in parenthesis in the axis label. **a** Plots PC-1 loading against PC-2. **b** Plots PC-1 against PC-3. **c** Plots PC-2 against PC-3. Red dots are main component of PC-1, blue dots are main components of PC-2, and orange dots are main components of PC-3. White dots indicate lower factor loading (< |0.3|). For the detail on factor loading scores, see Table 9

Table 9 shows the loadings on the first seven principal component (PC) variables which account for 64.6% of the variation in the original data. Figure 2 is the graphical representation of the loading on the first three PC variables which accounts for 32.40% of the total variation.

**Table 9:** Rotated factor loading of components identified by principal component analysis (PCA)

Interpreted biomarker component	Biomarker name	Factor loading^a^	Explained variance (%)	Explained variance cumulative (%)
PC-1	PFOS	0.853	12.27	12.27
	PFOA	0.696		
	PFOSA	0.523		
	Cu	0.452		
	AR-AEQ	− 0.386		
	ER-EEQ	0.363		
	Fe	0.358		
PC-2	AhR-TEQ	0.834	10.08	22.35
	Fe	0.758		
	AR-AEQ	0.658		
PC-3	I	0.763	10.05	32.40
	Zn	0.706		
	T3-ThEQ	0.589		
	Cr	0.427		
	Pb	0.326		
PC-4	Mn	0.777	8.43	40.83
	E2	0.763		
	ER-EEQ	0.347		
	Cr	0.316		
PC-5	Pb	0.778	8.34	49.17
	Cd	0.729		
	Cu	0.424		
	Cr	0.327		
PC-6	Se	0.746	7.76	56.93
	As	0.691		
	Cu	0.480		
PC-7	testosterone	0.818	7.71	64.63
	ER-EEQ	0.622		

*PC* principal component

^a^Factor loadings are the correlation coefficients between the original variables (levels of chemicals and receptor transactivities) and the extracted components. Variable levels are sorted by the size of the loading coefficients. Variable levels with factor loading below |0.30| are not listed

Summary statistics for the biomarkers with a detection frequency > 10% used in the PCA are found in the Additional file 6. Mann-Whitney test on the PCA scores for each component revealed that only the first principal component (PC-1), dominated by PFAS congeners, was significantly different between cases and controls (Additional file 7).

Logistic regression analysis showed a significantly negative association between ASD risk and first component (PC-1), including PFAS congeners, Cu, Fe, and ER-EEQ, both before and after adjustment for potential confounders (Table 10). After false discovery rate correction, the significance still persisted (adjusted OR = 0.100; 95%CI 0.016, 0.630; *p* = 0.014, FDR *q*_value_ = 0.098, Table 10). The other identified principal components did not show significant associations with ASD risk.

**Table 10:** Odds ratio and 95% confidence intervals for ASD according to PCA components

	Crude			Adjusted^a^			
	*n* (cases/controls)	OR (95% CI)	*p*	*n* (cases/controls)	OR (95% CI)	*p*	FDR (*q*_values_)
PC-1	29/44	*0.236 (0.092, 0.607)*	*0.003*	29/44	*0.100 (0.016, 0.630)*	*0.014*	*0.098*
PC-2	29/44	0.793 (0.449, 1.398)	0.422	29/44	0.943 (0.456, 1.948)	0.873	0.961
PC-3	29/44	1.180 (0.733, 1.897)	0.496	29/44	1.059 (0.538, 2.082)	0.868	0.868
PC-4	29/44	1.167 (0.724, 1.880)	0.526	29/44	1.027 (0.363, 2.903)	0.961	0.961
PC-5	29/44	1.064 (0.668, 1.695)	0.793	29/44	1.431 (0.810, 2.531)	0.218	0.763
PC-6	29/44	1.061 (0.664, 1.697)	0.804	29/44	1.258 (0.661, 2.393)	0.485	0.849
PC-7	29/44	1.25 (0.773, 2.025)	0.363	29/44	1.354 (0.662, 2.767)	0.407	0.849

*PC* principal component, *FDR (q*_*value*_*)* false discovery rate. For component of PC-1~PC-7, see Table 9. Italicized values indicate statistically significant (*p* < 0.05 and FDR *q*_value_ < 0.25)

^a^Adjusted for children’s birth year, children’s sex, mother age at delivery, father age at child birth, birth weight, gestational week at sampling, gestational age at birth, Apgar score, parity, and congenital malformation

After stratifying by children’s gender, the significant association of PC-1 and ASD risk disappeared which may due to reduced sample size (Additional file 8). When removing cases with congenital malformation, similar significant association between PC-1 and risk of ASD was found (Additional file 9).

## Discussion

The present study measured the levels of elements/metals and POPs such as PFAS in AF and receptor transactivities induced by AF, and explored their association with diagnoses of child ASD. We detected PFOS and PFOA in more than 46% of the AF samples and As, Cd, and Pb in up to 34% of the AF samples, suggesting that in addition to blood borne in utero exposure, the fetus also ingests and is exposed to low levels of PFAS and heavy metals. However, the lipophilic POPs, such as PCBs, PBDEs, and OCPs, were not detectable in AF. The combined biomarkers of effect such as ER-, AR-, AhR-, and TH-like activity were determined in the AF samples, indicating the presence of EDCs in amniotic fluid. Compared to the controls, ASD cases showed a different pattern regarding the AF levels of PFAS, heavy metals, and receptor activities. Although it lacks robust, statistically significant associations between EDCs and ASD risk, this study suggests a role of prenatal exposure to EDCs on the risk of ASD. But further studies are needed, especially for their effect interfering with the function of, e.g., sex hormone receptors such as ER and AR.

Stein et al. reported that PFOA and PFOS were detected in 86% and 32% of AF samples and suggested that PFOA was more commonly detected in AF than PFOS [76]. Similarly, in the present study, PFOA was detectable in 82% and PFOS was only detectable in 47% of the AF samples collected during 1995–1999, suggesting that most of the pregnant Danish women were exposed to both PFOA and PFOS. In the present study, the median PFOS level in AF sampling during 1995–1999 from male offspring pregnancies in the control group was 0.61 ng/ml, being lower than reported by Jensen et al. (1.1 ng/ml) in their samples collected during 1980–1996 [64], suggesting a decreasing PFOS exposure in Denmark after 1996. However, compared to the pregnant women in the USA during 2005–2008 [76], the AF level of PFOS in the control group in this study (1995–1999) was higher (1.44 vs 0.4 ng/ml) but similar for PFOA (0.32 vs 0.3 ng/ml), supporting an effective action of phase out of PFOS and PFOA in the USA [77]. The decreasing trend of PFOS concentrations observed in most European and North American studies follows the phasing out in 2000–2002 of perfluorooctanesulfonyl-fluoride-based (POSF) production by 3 M [78, 79]. Similarly, a decreasing trend for PFOA serum concentrations has also been observed in several studies, indicating that exposure pathways of these two compounds are historically linked [79, 80]. However, the production of PFOA precursor materials (ammonium perfluorooctanoate and fluorotelomer alcohols) has remained constant until 2006 [80]. Stein et al. did not detect PFOSA in the AF samples of pregnant women in the USA [76], while PFOSA was detectable in 35% of the AF samples in the present Danish population. PFOSA is a synthetic compound used to repel grease and water in food packing along with other consumer applications, and it breaks down to PFOS [81], which might partly explain the higher level of PFOS in the present study compared to pregnant women in the USA [76]. PFOSA was reported to have developmental neurotoxicity in vitro [82]. Although no obvious association was seen between PFOSA and ASD risk in this study, more research is warranted to study the effect of PFOSA.

The testosterone and estradiol were detectable in the AF samples. In ASD cases, the ratio of testosterone level over E2 level in AF was non-significantly higher compared to the controls. Baron-Cohen and co-workers also reported that autism cases had higher levels of testosterone and other precursor steroid hormones (progesterone, 17α-hydroxy-progesterone, and androstenedione) in the AF samples from same cohort (HBC) [14]. The fetal testosterone theory hypothesizes that higher levels of testosterone in AF pushes brain development towards improved ability to emphasize systemizing, which is a typical male trait and diminish female trait emphasizing [83]. An earlier study suggested that high levels of fetal testosterone could produce behaviors relevant to those observed for autism [84]. Auyeung et al. compared prenatal and postnatal testosterone and estradiol levels in relation to autistic traits in 18- to 24-month-old children. They found that prenatal (but not postnatal) androgen exposure, coinciding with the critical period for sexual differentiation of the brain, was associated with the development of autistic traits in 18- to 24-month-old toddlers. This suggests that prenatal, organizational effects of androgen hormones influence the development of autistic traits in later life [85]. Other studies reported that estrogen can be protective while androgens potentiate ASD [86, 87], supporting our observation of lower levels of endogenous estrogen E2 and higher endogenous androgen testosterone found in ASD cases. Furthermore, in support to our study, a higher ratio of testosterone to estradiol in AF was suggested to be associated with ASD [58],

EDCs deserve consideration as candidate risk factors for ASD because of their potential to alter hormonal axis functions that play an important role in neurodevelopment. However, few studies have examined the link between EDC exposure, especially for PFAS, and the risk of ASD or autistic behaviors. A cross-sectional study suggested that there might be a protective association between exposure to PFAS and cognition in older adults [88]. A prospective study showed better cognitive abilities among children with higher prenatal PFOA exposure [89], and increasing maternal blood PFOA concentrations were reported to be associated with fewer autistic behaviors [12]. Other studies did not find positive correlation between maternal PFAS exposure and attention-deficit hyperactivity disorder (ADHD) or ASD [90, 91]. Our previous study showed that PFAS, such as PFOS and PFOA, elicited estrogenic and anti-androgenic potential in vitro and ex vivo [23, 92]. In support, in the present study, we found PFAS levels positively correlated to the combined xenoestrogenic effect (EDC-EEQ) while inversely correlated to the combined xenoandrogenic effect (EDC-AEQ). The fetus seems to be uniquely sensitive to the anti-androgenic effect of EDCs. Given their anti-androgenic properties, one could hypothesize that PFAS exposure might reduce the risk of autism. This observation might partly explain the inverse association between PFAS level in AF and ASD risk observed in the present study. Moreover, in vitro studies report that PFOA and PFOS are partial agonists of the human peroxisome proliferator-activated receptor-γ (PPAR-γ), and PPAR-γ activation may be neuroprotective [93].

In the present AF study, we observed the ratio of AR-AEQ/ER-EEQ was non-significantly positively associated with ASD risk. AR-AEQ/ER-EEQ represents the proportion of total combined androgenic effect contributed by androgen and EDCs to the total combined estrogenic effect contributed by endogenous estrogen and EDCs. Hence, it is worthy to further study whether EDCs play a role in the development of ASD together with endogenous hormones.

Existing studies of autism suggest that prenatal metal exposure may be related to autism [94]. Exposure to Pb during neurodevelopment has significant effects on neurobehavioral and intellectual performance, also resulting in attention, hyperactivity, and learning disorders [17, 95, 96]. Lead poisoning has been suggested as a possible risk factor for autism, as Pb blood levels of autistic children have been reported to be significantly higher than those of healthy children [97]. Previous studies have shown that perinatal exposure to Mn, Pb, and Cd from air pollution increased ASD risk [42]. In the present study, we observed that As and Pb levels in AF tend to be positively associated with ASD risk, suggesting the possible role of prenatal exposure to toxic metals in the ASD development. However, given that these metals were detectable in less than 34% of the AF samples, more studies are needed to explore the role of prenatal heavy metal exposure on the risk of ASD.

There are several strengths in this study. First, the measurements of chemicals and receptor transactivities were based on the AF samples collected in pregnancy from routine amniocentesis prior to the assessment of the outcome in the offspring. Study participants were selected from a well-defined nation-wide pregnancy cohort and case-control matched by gender, year of birth, and maternal age. The outcome measures were clinical diagnoses using standardized diagnostic criteria (ICD classification), and diagnoses of autism in the psychiatric registry have been shown to have high validity [50]. Secondly, many exposure parameters were determined simultaneously in the single AF sample. This minimizes bias from differential misclassification. Thirdly, for the very first time, the combined mixture effect of EDCs was determined in AF. Moreover, the measurements of receptor transactivities consider the combined effect of EDC mixtures and endogenous hormones in AF. However, multiple testing issue should be addressed. We used the Benjamini-Hochberg method to control false discovery rate (FDR) and found that the significant association with the most individual exposure biomarker variables did not persist and thus cannot exclude a chance finding.

People are exposed to the mixture of different substances simultaneously, and therefore, it is important to assess the effect of actual combined effect of a mixture and principal component analysis (PCA) might provide the possibility to address this issue. The PCA extracts effective information from multidimensional data and is a powerful method to reduce the large number of highly correlated variables, so that results in fewer variables to be examined statistically. PCA reduced the false positive results by reducing the number of variables. Furthermore, PCA allows for the determination of which variables have common patterns of variances. PCA has been widely used for epidemiological studies [69]. To this aim, we further used PCA to extract the integrated principal component and analyzed the association of identified components and ASD risk. We observed a significantly negative association of ASD risk and the first principle component (PC-1), dominated by PFAS congeners and including Cu, Fe, and ER-EEQ, and the association persisted after corrected by FDR. Although it is difficult to interpret this observation clinically, this observation might suggest a role of combined effect of PFAS, Cu, Fe, and estrogenic EDCs on the risk of ASD and further studies are needed to explore the combined effect of substances which the fetus are exposed to simultaneously. It has been shown that the different PFAS congeners are highly correlated. Our previous studies have shown PFAS elicited the estrogenic effect and anti-androgenic effect [23, 92]. This was further confirmed by the result regarding the correlation of PFAS and receptor transactivities in the present study. Therefore, it is reasonable that PFAS congeners and ER-EEQ were identified in the same component. It is not clear why nutrients Cu and Fe were gathered together with PFAS and ER-EEQ. More studies are needed to explore whether it is because of similar exposure pathway.

In the present study, we included congenital malformation cases in the data set. Given the genetic factors that contributed ASD, we performed the sensitive analysis by further adjustment of congenital malformation and excluding cases with congenital malformation. The results did not change significantly because congenital malformation of cases and non-cases did not have significantly different levels in the exposure variables of interest.

This study has also some limitations. Firstly, the sample size in the present study was small due to toxic effect of rubber tube plugs used in connection with some of the cell culture analyses, and volume restriction for the parameters measured in each AF samples. The statistical power is thus expected to be low, and this might explain the lack of statistical significance. Because this study is the first study to explore the association of AF levels of PFAS and the combined EDC and endogenous hormone ex vivo receptor transactivities and ASD risk, it is hard to perform priori power calculation at the design stage. Nevertheless, we calculated the statistical power based on the average levels of exposure variables and the power ranged from 5.0 to 68.6% for individual exposure biomarkers. However, by using data reduction technique principal component analysis (PCA), the power is high for the first two identified principal components (PC-1 98.5%, PC-2 99.0%, respectively). Secondly, studies have reported that PFAS reduced fecundity in humans [98, 99]. Therefore, it is possible that PFAS exposure at a level that reduces fetal survival, especially in high-risk fetuses susceptible to neurological disorders such as ASD, could appear to have a null or even protective effect on adverse neurobehavioral outcomes in children in observational studies affected by selection bias, since only live born children can be followed-up and examined. All psychiatric and somatic diagnoses were retrieved from nation-wide registers (DPCR and DNHR), and no information on autistic clinical symptomatology was available to validate the diagnoses. However, other studies have shown that within the DPCR the validity ratio for childhood autism cases is 94.0% for 1990–1999 [50]. Thirdly, the measurements of chemicals and biomarkers of effects such as the combined (endogenous hormones and EDCs) induced receptor transactivities were done from a historic collection of the AF samples stored over several years at − 20 °C. However, for the cohort analyzed in this study (born between 1995 and 1999), storage time-dependent changes are unlikely to be a major issue [14]. The prevalence estimate of autism within this amniocentesis cohort (0.72%) is consistent with prevalence estimates of autism in the Danish population during the same time period [4]. Thus, although these results are derived from a selected sample, they may be representative of what would be expected in the general population. In the future, it is necessary to sample more representatives from the general population and collect more data regarding risk factors for fetal survival to further explore the integrated effect of EDCs on the development of autism. Finally, AF is an aqueous solution with suspended desquamated fetal epithelial cells. As gestation progresses, other organic materials including fetal feces and urine are also present. AF contains approximately equal parts of organics (proteins, carbohydrates, fats, enzymes, hormones, pigments) and inorganics. In human serum, some PFAS binds to circulating proteins (serum albumin) [100]. It is possible that some PFAS congeners were removed from the AF sample during its initial processing for clinical care purposes when the fluid was centrifuged and the precipitated cells retained for genetic testing [76]. Thus, the concentrations of PFAS in the AF samples might be underestimated and blur their effect on ASD.

## Conclusions and perspectives

The present study showed that environmental endocrine disrupting chemicals (EDCs), such as PFAS, some metals, and their biological activities can be detected in amniotic fluid, indicating that EDCs can cross the placenta and increase the potential fetal exposure to these environment contaminants. EDCs might modify ASD risk by influencing the hormone receptor function. The inverse association between PFAS levels and ASD risk might relate to a weak estrogenic activities and anti-androgenic activities of single PFAS congener observed in vitro. The observed tendency of positive association between the ratio of combined androgenic effect to the combined estrogenic effect and ASD risk needs further studies to explore the role of combined effect of EDCs together with endogenous hormones in the development of ASD. However, individual variability in genetic susceptibility can influence responses to environmental toxicants and contribute to increase disease vulnerabilities. Several studies have reported that some individuals with ASD express polymorphisms in genes involved in the detoxification of environmental pollutants and these genes have been termed “environmental response genes.” More than 100 environmental response genes may contribute to ASD risk [101, 102]. Therefore, it is necessary to study the role of gene polymorphisms and gene environment interaction in the development of ASD.

## Additional files

Additional file 1: The percentage of PFAS in the amniotic fluid samples which were above the detection limit. (DOCX 26 kb)

Additional file 2: The percentage of elements in the amniotic fluid samples which were above the detection limit. (DOCX 18 kb)

Additional file 3: Adjusted odds ratio and 95% confidence intervals for ASD according to chemicals in amniotic fluid stratified by gender. (DOCX 20 kb)

Additional file 4: Adjusted odds ratio and 95% confidence intervals for ASD according to chemicals, hormone level and receptor transactivity induced by amniotic fluid after removing congenital malformation. (DOCX 23 kb)

Additional file 5: Adjusted odds ratio and 95% confidence intervals for ASD according to hormone level and receptor transactivity induced by amniotic fluid stratified by gender. (DOCX 19 kb)

Additional file 6: Descriptive statistics for the exposure biomarkers with detection frequency > 10% in amniotic fluid and used in the principal component analysis. (DOCX 17 kb)

Additional file 7: Level of PCA scores. (DOCX 18 kb)

Additional file 8: Adjusted odds ratio and 95% confidence intervals for ASD according to PCA component stratified by gender. (DOCX 17 kb)

Additional file 9: Adjusted odds ratio and 95% confidence intervals for ASD according to PCA component after removing congenital malformation. (DOCX 17 kb)

## Abbreviations

ADHD: Attention-deficit hyperactivity disorder
AEQ: Androgen equivalent
AF: Amniotic fluid
AhR: Aryl hydrocarbon receptor
AhR-tact: Aryl hydrocarbon receptor transactivity
AR: Androgen receptor
AR-tact: Androgen receptor transactivity
ASD: Autism spectrum disorder
CD-FCS: Charcoal-dextran-treated fetal calf serum
DHT: Dihydrotestosterone
DMEM: Dulbecco’s modified Eagle medium
DNHR: Danish National Hospital Register
DPCR: Danish Psychiatric Central Register
E2: 17β-Estradiol
EDC-AEQ: EDC-induced androgenic effect
EDC-EEQ: EDC-induced estrogenic effect
EDCs: Endocrine disrupting compounds
EEQ: Estradiol equivalent
ER: Estrogen receptor
ER-tact: Estrogen receptor transactivity
FDR: False discovery rate
HBC: Historic Birth Cohort
ICD: International Classification of Diseases
OCP: Organchlorine pesticide
OR: Odds ratio
PBDE: Polybrominated diphenyl ether
PC: Principal component
PCA: Principal component analysis
PCB: Polychlorinated biphenyl
PE: Proliferation effect
PFAS: Perfluoroalkyl substances
PFBS: Perfluorobutane sulfonate
PFCA: Perfluorocarboxylated acids
PFDA: Perfluorodecanoic acid
PFDoA: Perfluorododecanoic acid
PFDS: Perfluorodecane sulfonate
PFHpA: Perfloroheptanoate
PFHpS: Perfluoroheptane sulfonate
PFHxA: Perfluorohexanoic acid
PFHxS: Perfluorohexane sulfonate
PFNA: Perfluorononanoic acid
PFOA: Perfluorooctanoic acid
PFOS: Perfluorooctane sulfonate
PFOSA: Perfluorooctane sulfonamide
PFPeA: Perfluoro-n-pentanoic acid
PFSA: Perfluorosulfonated acids
PFUnA: Perfluoroundecanoic acid
POP: Persistent organic pollutants
POSF: Perfluorooctanesulfonyl-fluoride
PPAR: Proliferator-activated receptor
T3: Triiodothyronine
TCDD: 2, 3, 7, 8-Tetrachlorodibenzo-p-dioxin
TEQ: TCDD equivalent
TH: Thyroid hormone
ThEQ: Thyroid equivalent
TH-PE: Thyroid hormone-related proliferative effect
