# Supplementary material for: Autism spectrum disorders, endocrine disrupting compounds, and heavy metals in amniotic fluid: a case-control study
Source: Mol Autism. 2019 Jan 9;10:1. doi: 10.1186/s13229-018-0253-1 (PMC6327542; doi:10.1186/s13229-018-0253-1)
Supplement: Supplementary file 1 — The percentage of PFAS in the amniotic fluid samples which were above the detection limit. (DOCX 26 kb) [file 13229_2018_253_MOESM1_ESM.docx]

**Additional file 1.** The percentage of PFAS in the amniotic fluid samples which were above the detection limit

| PFAS congeners | Limit of quantification (LOQ)  ng/mL | % of above LOQ |
| --- | --- | --- |
| PFOA | 0.20 | 81.80 |
| PFOS | 1.22 | 46.60 |
| PFOSA | 1.19 | 35.20 |
| PFHxS | 0.13 | 4.50 |
| PFDoA | 0.41 | 1.10 |
| PFHpS | 0.14 | 0 |
| PFBS | 0.07 | 0 |
| PFDS | 0.37 | 0 |
| PFPeA | 0.31 | 0 |
| PFHxA | 0.51 | 0 |
| PFHpA | 0.05 | 0 |
| PFNA | 0.27 | 0 |
| PFDA | 0.20 | 0 |
| PFUnA | 0.75 | 0 |
