# Supplementary material for: Autism spectrum disorders, endocrine disrupting compounds, and heavy metals in amniotic fluid: a case-control study
Source: Mol Autism. 2019 Jan 9;10:1. doi: 10.1186/s13229-018-0253-1 (PMC6327542; doi:10.1186/s13229-018-0253-1)
Supplement: Supplementary file 2 — The percentage of elements in the amniotic fluid samples which were above the detection limit. (DOCX 18 kb) [file 13229_2018_253_MOESM2_ESM.docx]

**Additional file 2.** The percentage of elements in the amniotic fluid samples which were above the detection limit

| **Element** | **Detection Limit (DL)**  **µg/L** | **% over DL** |  | **Element** | **Detection Limit (DL)**  **µg/L** | **% over DL** |
| --- | --- | --- | --- | --- | --- | --- |
| **Li** | 0.23 | 100 |  | **Cd** | 0.04 | 12.5 |
| **Na** | 9075 | 100 |  | **Gd** | 0.01 | 12.5 |
| **Mg** | 31.60 | 100 |  | **Y** | 0.01 | 12.5 |
| **P** | 318 | 100 |  | **Pt** | 0.34 | 11.4 |
| **S** | 9253 | 100 |  | **La** | 0.01 | 9.10 |
| **Cl** | 159970 | 100 |  | **Sm** | 0.01 | 8.00 |
| **K** | 510 | 100 |  | **Ti** | 0.97 | 8.0 |
| **Ca** | 28.00 | 100 |  | **Al** | 4.57 | 6.8 |
| **Fe** | 16.40 | 100 |  | **Tl** | 0.66 | 5.70 |
| **Cu** | 0.59 | 100 |  | **Ce** | 0.01 | 5.70 |
| **Zn** | 1.16 | 100 |  | **Pr** | 0.005 | 4.50 |
| **Se** | 0.78 | 100 |  | **Tb** | 0.004 | 3.40 |
| **Br** | 8.37 | 100 |  | **Er** | 0.01 | 3.40 |
| **Rb** | 0.38 | 100 |  | **Nd** | 0.02 | 2.30 |
| **Sr** | 0.16 | 100 |  | **Sc** | 0.12 | 2.30 |
| **Cs** | 0.04 | 100 |  | **Eu** | 0.01 | 1.10 |
| **Cr** | 0.31 | 98.9 |  | **Dy** | 0.01 | 1.10 |
| **Si** | 3.79 | 93.2 |  | **Tm** | 0.004 | 1.10 |
| **Mo** | 0.73 | 76.1 |  | **Yb** | 0.02 | 1.10 |
| **V** | 0.15 | 65.9 |  | **Bi** | 0.03 | 1.10 |
| **Ni** | 1.17 | 48.9 |  | **U** | 0.02 | 1.10 |
| **Ba** | 0.39 | 34.1 |  | **Hg** | 1.29 | 0 |
| **Pb** | 0.44 | 34.1 |  | **Ag** | 0.41 | 0 |
| **I** | 3.76 | 33.0 |  | **Te** | 0.30 | 0 |
| **As** | 2.66 | 22.7 |  | **Sn** | 1.04 | 0 |
| **Zr** | 0.70 | 20.5 |  | **Sb** | 1.28 | 0 |
| **Mn** | 1.29 | 18.2 |  | **Th** | 0.02 | 0 |
| **Ho** | 0.003 | 18.2 |  | **Co** | 0.91 | 0 |
| **Lu** | 0.003 | 17.0 |  | **Ga** | 0.04 | 0 |
| **Be** | 0.02 | 15.9 |  |  |  |  |
