# Supplementary material for: Autism spectrum disorders, endocrine disrupting compounds, and heavy metals in amniotic fluid: a case-control study
Source: Mol Autism. 2019 Jan 9;10:1. doi: 10.1186/s13229-018-0253-1 (PMC6327542; doi:10.1186/s13229-018-0253-1)
Supplement: Supplementary file 3 — Adjusted odds ratio and 95% confidence intervals for ASD according to chemicals in amniotic fluid stratified by gender. (DOCX 20 kb) [file 13229_2018_253_MOESM3_ESM.docx]

Additional file 3. Adjusted Odds ratio and 95% confidence intervals for ASD according to chemicals in amniotic fluid stratified by gender

|  | Male | | | |  | Female | | | |
| --- | --- | --- | --- | --- | --- | --- | --- | --- | --- |
|  | n (cases/controls) | OR (95% CI) | *p* | FDR (q_value_) |  | n (cases/controls) | OR (95% CI) | *p* | *FDR (q_value_)* |
| PFOS (ng/mL) | 29/35 | 0.586(0.192;1.782) | 0.346 | 0.536 |  | 7/15 | 0.027(0;4.755) | 0.171 | 0.576 |
|  |  |  |  |  |  |  |  |  |  |
| PFOSA (ng/mL) | 29/35 | 0.834(0.631;1.104) | 0.205 | 0.536 |  | 7/15 | 0.952(0.751;1.207) | 0.684 | 0.897 |
|  |  |  |  |  |  |  |  |  |  |
| PFOA (ng/mL) | 29/35 | 0.270(0.020;3.634) | 0.323 | 0.536 |  | 7/15 | 0.001(0;192.7)^b^ | 0.275 | 0.675 |
|  |  |  |  |  |  |  |  |  |  |
| ∑PFSA (ng/mL) | 29/35 | 0.836(0.644;1.086) | 0.179 | 0.536 |  | 7/15 | 0.928(0.730;1.181) | 0.545 | 0.818 |
|  |  |  |  |  |  |  |  |  |  |
| ∑PFCA (ng/mL) | 29/35 | 0.270(0.020;3.634) | 0.323 | 0.536 |  | 7/15 | 0.002(0;443.4) | 0.327 | 0.679 |
|  |  |  |  |  |  |  |  |  |  |
| ∑PFSA+∑PFCA (ng/mL) | 29/35 | 0.835(0.648;1.075) | 0.162 | 0.536 |  | 7/15 | 0.924(0.727;1.175) | 0.519 | 0.818 |
|  |  |  |  |  |  |  |  |  |  |
| Fe (µg/L) | 29/35 | 1.000(0.997;1.003) | 0.891 | 0.891 |  | 7/15 | 0.999(0.993;1.006) | 0.857 | 0.968 |
|  |  |  |  |  |  |  |  |  |  |
| Cu (µg/L) | 29/35 | 1.004(0.983;1.027) | 0.690 | 0.745 |  | 7/15 | 1.006(0.961;1.053) | 0.803 | 0.968 |
|  |  |  |  |  |  |  |  |  |  |
| Zn (µg/L) | 29/35 | 0.998(0.992;1.004) | 0.535 | 0.676 |  | 7/15 | 1.003(0.994;1.011) | 0.541 | 0.818 |
|  |  |  |  |  |  |  |  |  |  |
| Se (µg/L) | 29/35 | 1.134(0.624;2.061) | 0.680 | 0.745 |  | 7/15 | 9.710(0.342;275.3) | 0.183 | 0.576 |
|  |  |  |  |  |  |  |  |  |  |
| I (µg/L) | 29/35 | *0.973 (0.948;0.999)* | *0. 042* | 0.536 |  | 7/15 | 6.4x10^17^ (0;~) | 0.991 | 0.999 |
|  |  |  |  |  |  |  |  |  |  |
| Cr (µg/L) | 29/35 | 0.403(0.031;5.238) | 0.487 | 0.657 |  | 7/15 | 0.002(0;62.976) | 0.243 | 0.619 |
|  |  |  |  |  |  |  |  |  |  |
| Mn (µg/L) | 29/35 | 1.197(0.822;1.743) | 0.348 | 0.536 |  | 7/15 | 0.483(0.012;19.051) | 0.698 | 0.897 |
|  |  |  |  |  |  |  |  |  |  |
| As (µg/L) | 29/35 | 1.402(0.767;2.562) | 0.272 | 0.536 |  | 7/15 | 2.631(0.666;10.390) | 0.167 | 0.576 |
|  |  |  |  |  |  |  |  |  |  |
| Cd (µg/L) | 29/35 | 11.177(0.065;1909) | 0.357 | 0.536 |  | 7/15 | 0(0;~) | 0.999 | 0.999 |
|  |  |  |  |  |  |  |  |  |  |
| Pb (µg/L) | 29/35 | 1.685(0.762;3.726) | 0.197 | 0.536 |  | 7/15 | 0.691(0.011;42.203) | 0.860 | 0.968 |

OR was obtained from the continuous variables. Adjusted for children’s birth year, mother age at delivery, father age at child birth, birth weight, gestational week at sampling, gestational age at birth, Apgar score, parity and congenital malformation. FDR (q_value_): false discovery rate. Italicized values indicates statistically significant (p <0.05, FDR q_value_ < 0.25).
