# Supplementary material for: Autism spectrum disorders, endocrine disrupting compounds, and heavy metals in amniotic fluid: a case-control study
Source: Mol Autism. 2019 Jan 9;10:1. doi: 10.1186/s13229-018-0253-1 (PMC6327542; doi:10.1186/s13229-018-0253-1)
Supplement: Supplementary file 4 — Adjusted odds ratio and 95% confidence intervals for ASD according to chemicals, hormone level and receptor transactivity induced by amniotic fluid after removing congenital malformation. (DOCX 23 kb) [file 13229_2018_253_MOESM4_ESM.docx]

Additional file 4. Adjusted odds ratio and 95% confidence intervals for ASD according to chemicals, hormone level and receptor transactivity induced by amniotic fluid after removing congenital malformation

|  | n (cases/controls) | OR (95% CI) | *p* | *FDR (q_value_)* |
| --- | --- | --- | --- | --- |
| PFOS (ng/mL) | 36/50 | *0.409 (0.176;0.951)* | *0.038* | 0.483 |
|  |  |  |  |  |
| PFOSA (ng/mL) | 36/50 | 0.914 (0.784;1.065) | 0.249 | 0.528 |
|  |  |  |  |  |
| PFOA (ng/mL) | 36/50 | 0.155 (0.012;2.051) | 0.157 | 0.483 |
|  |  |  |  |  |
| ∑PFSA (ng/mL) | 36/50 | 0.895 (0.766;1.045) | 0.161 | 0.483 |
|  |  |  |  |  |
| ∑PFCA (ng/mL) | 36/50 | 0.141 (0.012;1.686) | 0.122 | 0.483 |
|  |  |  |  |  |
| ∑PFSA+∑PFCA (ng/mL) | 36/50 | 0.891 (0.764;1.040) | 0.142 | 0.483 |
|  |  |  |  |  |
| Fe (µg/L) | 36/50 | 0.999 (0.997;1.002) | 0.456 | 0.648 |
|  |  |  |  |  |
| Cu (µg/L) | 36/50 | 0.996 (0.981;1.011) | 0.607 | 0.712 |
|  |  |  |  |  |
| Zn (µg/L) | 36/50 | 0.999 (0.995;1.003) | 0.575 | 0.712 |
|  |  |  |  |  |
| Se (µg/L) | 36/50 | 1.061 (0.646;1.742) | 0.815 | 0.815 |
|  |  |  |  |  |
| I (µg/L) | 36/50 | 0.986 (0.965;1.007) | 0.183 | 0.494 |
|  |  |  |  |  |
| Cr (µg/L) | 36/50 | 0.309 (0.044;2.145) | 0.235 | 0.528 |
|  |  |  |  |  |
| Mn (µg/L) | 36/50 | 1.092 (0.868;1.373) | 0.453 | 0.648 |
|  |  |  |  |  |
| As (µg/L) | 36/50 | 1.406 (0.874;2.263) | 0.160 | 0.483 |
|  |  |  |  |  |
| Cd (µg/L) | 36/50 | 5.908 (0.099;353.8) | 0.395 | 0.631 |
|  |  |  |  |  |
| Pb (µg/L) | 36/50 | 1.235 (0.627;2.429) | 0.542 | 0.712 |
|  |  |  |  |  |
| E2 (pg/mL ) | 65/123 | 1.000(0.999;1.002) | 0.587 | 0.712 |
|  |  |  |  |  |
| Testerone (pg/mL ) | 58/104 | *1.002(1.000;1.004)* | *0.045* | 0.483 |
|  |  |  |  |  |
| Testerone/E2 | 63/123 | 1.283(0.914;1.801) | 0.150 | 0.483 |
|  |  |  |  |  |
| ER-EEQ (pg E2/mL) | 71/130 | 1.000(0.999;1.000) | 0.397 | 0.631 |
|  |  |  |  |  |
| EDCs- EEQ (pg E2/mL) | 65/123 | 0.999(0.998;1.000) | 0.152 | 0.483 |
|  |  |  |  |  |
| AR-AEQ (pg DHT/mL) | 71/130 | 0.997(0.986;1.009) | 0.633 | 0.712 |
|  |  |  |  |  |
| EDCs- AEQ (pg DHT/mL) | 64/125 | 0.992(0.978;1.006) | 0.254 | 0.528 |
|  |  |  |  |  |
| AR-AEQ/ER-EEQ | 71/130 | 1.811(0.113;29.002) | 0.675 | 0.729 |
|  |  |  |  |  |
| EDCs-AEQ/EDCs-EEQ | 63/123 | 1.024(0.969;1.083) | 0.393 | 0.631 |
|  |  |  |  |  |
| T3-ThEQ (ng T3/mL) | 71/128 | 1.126(0.889;1.424) | 0.325 | 0.627 |
|  |  |  |  |  |
| AhR-TEQ (pgTCDD/mL) | 36/50 | 0.829(0.201; 3.414) | 0.795 | 0.815 |

OR was obtained from the continuous variables. Adjusted for children’s birth year, children’s sex, mother age at delivery, father age at child birth, birth weight, gestational age at birth, gestational week at sampling, Apgar score, parity.

E2: 17β-Estradiol; ER-EEQ: The combined effect on ER of endogenous estrogen and xeno-estrogens (EDCs) given as estradiol equivalent. EDC- EEQ: The integrated estrogenic effect induced only by xeno-estrogens (EDCs) alone given as estradiol equivalent; DHT: Dihydrotestosterone; AR-AEQ: The combined effect on AR of endogenous androgens and xeno-androgens (EDCs) given as androgen equivalent; EDC-AEQ: The integrated androgenic effect induced only by xeno-androgens (EDCs) alone given as androgen equivalent; T3: L-3,5,3’-Triiiodothyronine; T3-ThEQ: The combined proliferation effect (PE) of endogenous thyroid and xeno-thyroid hormone like compounds (EDCs) given as thyroid equivalent; AhR-TEQ: The combined effect on AhR of dioxin-like compounds given as TCDD equivalent. FDR (q_value_): false discovery rate. Italicized values indicates statistically significant (p <0.05, FDR q_value_ < 0.25).
