# Supplementary material for: Autism spectrum disorders, endocrine disrupting compounds, and heavy metals in amniotic fluid: a case-control study
Source: Mol Autism. 2019 Jan 9;10:1. doi: 10.1186/s13229-018-0253-1 (PMC6327542; doi:10.1186/s13229-018-0253-1)
Supplement: Supplementary file 6 — Descriptive statistics for the exposure biomarkers with detection frequency > 10% in amniotic fluid and used in the principal component analysis. (DOCX 17 kb) [file 13229_2018_253_MOESM6_ESM.docx]

**Additional file 6.** Descriptive statistics for the exposure biomarkers with detection frequency > 10% in amniotic fluid and used in the principal component analysis

|  | n | Median | Mean | SD | Min | Max |  |
| --- | --- | --- | --- | --- | --- | --- | --- |
| PFOS (ng/mL) | 88 | 0.61 | 1.19 | 0.76 | 0.61 | 4.22 |  |
| PFOSA(ng/mL) | 88 | 0.60 | 2.23 | 3.94 | 0.60 | 19.00 |  |
| PFOA(ng/mL) | 88 | 0.31 | 0.35 | 0.25 | 0.10 | 1.86 |  |
| Fe (µg/L) | 88 | 260.50 | 330.23 | 302.94 | 16.00 | 2575.00 |  |
| Cu (µg/L) | 88 | 91.76 | 100.63 | 33.85 | 45.62 | 223.05 |  |
| Zn (µg/L) | 88 | 101.96 | 137.43 | 152.91 | 30.90 | 1215.49 |  |
| Se (µg/L) | 88 | 4.51 | 4.62 | 1.04 | 2.25 | 7.27 |  |
| I (µg/L) | 88 | 1.88 | 9.51 | 25.47 | 1.88 | 195.44 |  |
| Cr (µg/L) | 88 | 0.74 | 0.83 | 0.46 | 0.16 | 3.85 |  |
| Mn (µg/L) | 88 | 0.65 | 1.24 | 2.79 | 0.65 | 25.89 |  |
| As (µg/L) | 88 | 1.33 | 1.88 | 1.12 | 1.33 | 6.19 |  |
| Cd (µg/L) | 88 | 0.02 | 0.05 | 0.20 | 0.02 | 1.92 |  |
| Pb (µg/L) | 88 | 0.22 | 0.57 | 0.74 | 0.22 | 5.30 |  |
| E2 (pg/mL) | 196 | 303.70 | 345.30 | 254.54 | 55.02 | 2778.28 |  |
| Testosterone (pg/mL) | 197 | 374.95 | 407.44 | 214.78 | 47.30 | 1393.07 |  |
| ER-EEQ (pgE2/mL) | 210 | 500.75 | 705.11 | 429.36 | 149.00 | 1307.50 |  |
| AR-AEQ (pgDHT/mL) | 210 | 62.60 | 67.08 | 28.78 | 16.90 | 290.40 |  |
| T3-ThEQ (ngT3/mL) | 207 | 2.39 | 2.56 | 1.29 | 0 | 17.14 |  |
| AhR-TEQ(pg TCDD/mL) | 88 | 0.19 | 0.29 | 0.37 | 0 | 2.10 |  |
