# Supplementary material for: Autism spectrum disorders, endocrine disrupting compounds, and heavy metals in amniotic fluid: a case-control study
Source: Mol Autism. 2019 Jan 9;10:1. doi: 10.1186/s13229-018-0253-1 (PMC6327542; doi:10.1186/s13229-018-0253-1)
Supplement: Supplementary file 7 — Level of PCA scores. (DOCX 18 kb) [file 13229_2018_253_MOESM7_ESM.docx]

**Additional file 7.** Level of PCA scores

| Principal component | ASD cases (n=29) | |  | Control (n=44) | | *p* |  |
| --- | --- | --- | --- | --- | --- | --- | --- |
|  | Median | Min; max |  | median | Min; max |  | FDR(q_value_) |
| PC-1 | *-0.47* | *-1.64;1.56* |  | *0.06* | *-1.08; 4.03* | *<0.0001* | *0.0007* |
| PC-2 | -0.30 | -0.79;1.41 |  | -0.02 | -1.70;6.35 | 0.752 | 0.752 |
| PC-3 | -0.02 | -1.41;5.95 |  | -0.27 | -1.30;2.71 | 0.535 | 0.752 |
| PC-4 | -0.16 | -1.52;6.36 |  | -0.05 | -1.32;1.73 | 0.668 | 0.752 |
| PC-5 | -0.30 | -1.05;5.92 |  | -0.12 | -1.04;3.82 | 0.232 | 0.752 |
| PC-6 | -0.19 | -1.44;3.38 |  | -0.06 | -1.39;2.59 | 0.604 | 0.752 |
| PC-7 | -0.03 | -1.28;1.82 |  | 0.02 | -3.11;2.00 | 0.423 | 0.752 |

PC: principal component. Mann-Whitney test was used to compare the difference between ASD cases and controls. FDR (q_value_): false discovery rate. Italicized values indicates statistically significant (p <0.05, FDR q_value_ < 0.25)
