# Supplementary material for: Autism spectrum disorders, endocrine disrupting compounds, and heavy metals in amniotic fluid: a case-control study
Source: Mol Autism. 2019 Jan 9;10:1. doi: 10.1186/s13229-018-0253-1 (PMC6327542; doi:10.1186/s13229-018-0253-1)
Supplement: Supplementary file 8 — Adjusted odds ratio and 95% confidence intervals for ASD according to PCA component stratified by gender. (DOCX 17 kb) [file 13229_2018_253_MOESM8_ESM.docx]

Additional file 8. Adjusted odds ratio and 95% confidence intervals for ASD according to PCA component stratified by gender

|  | Male | | | |  | Female | | | |
| --- | --- | --- | --- | --- | --- | --- | --- | --- | --- |
|  | n (cases/controls) | OR (95% CI) | *p* | *FDR (q_value_)* |  | n (cases/controls) | OR (95% CI) | *p* | *FDR (q_value_)* |
| PC-1 | 25/31 | 0.189(0.026;1.376) | 0.100 | 0.472 |  | 4/12 | 0 | 0.999 | 0.999 |
|  |  |  |  |  |  |  |  |  |  |
| PC-2 | 25/31 | 1.601(0.655;3.911) | 0.302 | 0.551 |  | 4/12 | 0 | 0.999 | 0.999 |
|  |  |  |  |  |  |  |  |  |  |
| PC-3 | 25/31 | 1.310(0.424;4.050) | 0.639 | 0.848 |  | 4/12 | 0 | 1.000 | 0.999 |
|  |  |  |  |  |  |  |  |  |  |
| PC-4 | 25/31 | 1.080(0.282;4.161) | 0.911 | 0.911 |  | 4/12 | 5.35x10^6^ | 0.999 | 0.999 |
|  |  |  |  |  |  |  |  |  |  |
| PC-5 | 25/31 | 1.639(0.857;3.133) | 0.135 | 0.473 |  | 4/12 | 1.772x10^13^ | 0.999 | 0.999 |
|  |  |  |  |  |  |  |  |  |  |
| PC-6 | 25/31 | 1.147(0.531;2.476) | 0.727 | 0.848 |  | 4/12 | 1.430x10^9^ | 0.999 | 0.999 |
|  |  |  |  |  |  |  |  |  |  |
| PC-7 | 25/31 | 1.542(0.662;3.589) | 0.315 | 0.551 |  | 4/12 | 2.265x10^5^ | 0.999 | 0.999 |
|  |  |  |  |  |  |  |  |  |  |

PC: principal component. OR was obtained from the continuous variables. Adjusted for children’s birth year, mother age at delivery, father age at child birth, birth weight, gestational age at birth, gestational week at sampling, Apgar score, parity and congenital malformation. FDR (q_value_): false discovery rate. .
