# Supplementary material for: Autism spectrum disorders, endocrine disrupting compounds, and heavy metals in amniotic fluid: a case-control study
Source: Mol Autism. 2019 Jan 9;10:1. doi: 10.1186/s13229-018-0253-1 (PMC6327542; doi:10.1186/s13229-018-0253-1)
Supplement: Supplementary file 9 — Adjusted odds ratio and 95% confidence intervals for ASD according to PCA component after removing congenital malformation. (DOCX 17 kb) [file 13229_2018_253_MOESM9_ESM.docx]

Additional file 9. Adjusted odds ratio and 95% confidence intervals for ASD according to PCA component after removing congenital malformation

|  | n (cases/controls) | OR (95% CI) | *p* | *FDR (q_value_)* |
| --- | --- | --- | --- | --- |
| PC-1 | 24/43 | *0.100(0.016;0.630)* | *0.014* | *0.098* |
|  |  |  |  |  |
| PC-2 | 24/43 | 0.943(0.456;1.948) | 0.873 | 0.961 |
|  |  |  |  |  |
| PC-3 | 24/43 | 1.059(0.538;2.082) | 0.868 | 0.961 |
|  |  |  |  |  |
| PC-4 | 24/43 | 1.027(0.363;2.903) | 0.961 | 0.961 |
|  |  |  |  |  |
| PC-5 | 24/43 | 1.431(0.810;2.531) | 0.218 | 0.763 |
|  |  |  |  |  |
| PC-6 | 24/43 | 1.258(0.661;2.393) | 0.485 | 0.849 |
|  |  |  |  |  |
| PC-7 | 24/43 | 1.354(0.662;2.767) | 0.407 | 0.849 |
|  |  |  |  |  |

PC: principal component. OR was obtained from the continuous variables. Adjusted for children’s birth year, children’s sex, mother age at delivery, father age at child birth, birth weight, gestational age at birth, gestational week at sampling, Apgar score, parity. FDR (q_value_): false discovery rate. Italicized values indicates statistically significant (p <0.05, FDR q_value_ < 0.25) .
